# Structural Quality Factor of Flo‐TENG under Stochastic Wave Excitation

**DOI:** 10.1002/advs.202405165

**Published:** 2024-08-09

**Authors:** Dongxin Guo, Chunjin Chen, Jiawei Li, Lixia Zhai, Songying Li, Sheng He, Junrui Feng, Lingyu Wan, Guanlin Liu, Junyi Zhai

**Affiliations:** ^1^ Center on Nanoenergy Research Institute of Science and Technology for Carbon Peak & Neutrality State Key Laboratory of Featured Metal Materials and Life‐cycle Safety for Composite Structures School of Physical Science & Technology Guangxi University Nanning 530004 China; ^2^ Guangxi Key Laboratory for the Relativistic Astrophysics Guangxi University Nanning 530004 China; ^3^ CAS Center for Excellence in Nanoscience Beijing Key Laboratory of Micro‐nano Energy and Sensor Beijing Institute of Nanoenergy and Nanosystems Chinese Academy of Sciences Beijing 101400 China; ^4^ School of Nanoscience and Engineering University of Chinese Academy of Sciences Beijing 100049 China

**Keywords:** floating triboelectric nanogenerator, stochastic wave excitation, structural quality factor

## Abstract

Triboelectric nanogenerators (TENGs) have recently emerged as a promising technology for efficient water wave energy harvesting. However, there is a paucity of clear guidance regarding the optimal designs of TENGs and their shells to achieve efficient absorption and conversion of water wave energy in real random waves. Herein, from the perspective of wave‐body interaction and energy transfer, this paper proposes a structural quality factor (Q_unit_) for the quantitative evaluation of both the motion of floating triboelectric nanogenerator (Flo‐TENG) shells and their capability to absorb and convert water wave energy efficiently. The factor is further subdivided into the amplitude structural quality factor (Q_acc_), which characterizes shell motion amplitude, and the frequency structural quality factor (Q_f_), which describes shell motion frequency. This paper systematically investigates the impact of various shell parameters such as bow shapes, curvatures, inclinations, and immersion ratios on Q_acc_ and Q_f_. The findings indicate that variations in shell shape result in distinct Q_unit_ values along different axial directions of wave propagation. These variations directly influence energy absorption efficiency in these directions. These results provide fundamental guidance for the design of high‐performance Flo‐TENG shells and the selection of internal energy harvesting directions to enable more efficient energy conversion.

## Graphical abstract

This study proposes a quality factor for quantitatively assessing the motion of floating TENG shells and their effective absorption and conversion of wave energy. The influence of various parameters is systematically investigated, leading to the identification of several patterns. These findings provide guidance for designing high‐performance floating TENG shells and selecting internal energy harvesting directions, enabling more efficient energy conversion.

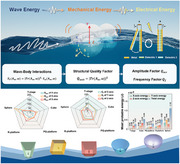

## 1 Introduction

With the advancement of marine informatics and the Internet of Things (IoTs),^[^ ^1^, ^2^, ^3^ ^]^ the proliferation of distributed sensors at sea has significantly intensified the urgent need for powering sources.^[^ ^4^, ^5^, ^6^, ^7^, ^8^ ^]^ Solar energy, commonly utilized in maritime settings, faces challenges in insufficient light energy during night scenarios and prolonged overcast and rainy weather, rendering them ineffective in generating substantial electrical output.^[^ ^9^ ^]^ In contrast, widespread distributed wave energy on the sea is abundant and inexhaustible, serving as a clean sustainable energy source.^[^ ^10^, ^11^, ^12^ ^]^ Triboelectric nanogenerators (TENGs) based on displacement current offer an effective solution for harnessing such high‐entropy, low‐frequency mechanical energy.^[^ ^13^, ^14^ ^]^ As opposed to the currently developed wave energy harvesting technologies based on electromagnetic generators, TENGs have the advantages of simple fabrication, a wide choice of materials, and high energy conversion efficiency at low frequencies. Based on these unique advantages, TENGs have rapidly become a promising technology in the field of wave energy harvesting.^[^ ^15^, ^16^, ^17^ ^]^

In recent years, various shapes and structures of TENGs including spherical,^[^ ^18^, ^19^, ^20^, ^21^ ^]^ oblate spheroidal,^[^ ^22^, ^23^, ^24^ ^]^ cylindrical,^[^ ^25^, ^26^ ^]^ boat‐shaped,^[^ ^27^, ^28^, ^29^ ^]^ flower‐like,^[^ ^30^ ^]^ tensegrity‐based^[^ ^31^ ^]^ and pendulum‐based^[^ ^32^, ^33^ ^]^ designs have been proposed for efficient wave energy harvesting. However, there is currently a lack of clear guidance on the optimal design of an ocean kinetic energy harvester (OKEH) and its floating shell.^[^ ^34^, ^35^ ^]^ Specifically, a standardized approach for quantitatively assessing the energy capture performance of OKEH remains a challenge under random wave excitation.^[^ ^36^, ^37^, ^38^, ^39^ ^]^ In the existing performance evaluation system for TENGs, the figure of merit (*FOM_p_*) of a triboelectric nanogenerator (TENG) unit under a defined rule excitation is defined as $FOM_{p}=\;2\varepsilon_{0}\frac{E_{m}}{Ax_{max}}$, which is collectively determined by the surface area *A* of the triboelectric layer, the maximum displacement *x_max_* of the moving unit, and the output energy *E_m_* within a single motion cycle.^[^ ^40^ ^]^ Whereas, triggered by random fluctuating excitation of ocean waves, the output of TENG exhibits typical characteristics of random signals, wherein a single moving cycle fails to fully reflect its overall performance.^[^ ^20^, ^41^, ^42^ ^]^ Furthermore, in marine environments, TENG units are typically encased within shells of specific shapes, where The electrical energy output is dependent on two factors: the efficiency of the TENG in converting the kinetic energy of the motion into electricity and the efficiency of the shell in absorbing wave energy.^[^ ^43^ ^]^ Unfortunately, the factor *FOM_p_* does not account for the influence of the shell on wave energy absorption. In 2022, Xu et al. demonstrated that the motion of the shell under wave excitation is closely related to the geometric parameters and the center of mass. Nevertheless, the study did not account for the stochastic nature of waves and did not provide specific evaluation methodologies. Hence, it is imperative to develop a universal approach for assessing the performance of TENG with shells under random external excitation. On one hand, the consideration of statistical characteristics within a stochastic process is necessary when analyzing the output of a TENG. On the other hand, the mechanisms by which individual physical quantities related to the shape of the shell play a role in the wave‐body coupling process must be thoroughly investigated.

In this paper, we present a quantitative method for evaluating the energy absorption performance of floating TENGs by employing statistical analysis and investigating the energy transfer relationship. Initially, wave spectral analysis is introduced to describe the energy transfer relationship governing wave energy harvesting and electrical power. Based on the transfer function, we propose a structural quality factor (*Q_unit_*) for assessing the structural quality factor of TENG shells, which comprises amplitude structure quality factor (*Q_acc_*) and frequency structure quality factor (*Q_f_*). Furthermore, we systematically investigated the effect of physical quantities related to shell shape on *Q_unit_* in a real marine environment. In experiments, we integrated inertial measurement units into floating shells with varying bow shapes, wavefront curvatures, inclination angles, and immersion ratios to acquire acceleration data. Concurrently, we also measured the wave spectrum parameters in the experimental sea area using a wave gauge. Through spectral analysis and calculations, we obtained probability density distributions of acceleration amplitude and frequency, radar plots illustrating *Q_acc_* and *Q_f_*, and histograms describing the most probable energy values (*E_mp_*), which reflect the magnitude of energy absorbed along each axis. The results visualize the performance of different shells in terms of energy absorption and transfer and reveal the effect of shape parameters on *Q_acc_* and Q_f_. It has been observed that different shape parameters have different mechanisms of action in energy absorption and transfer. The variation of each parameter will cause changes in the energy transfer and absorption performance in different axial directions of the device. This evidence demonstrates that the absorption and transformation of energy by Flo‐TENG during the performance of wave‐body interaction is axial in nature. And the *Q_unit_* is a reliable quantitative measure of the changes in the energy absorption and transformation performance of different devices in each axial direction. Consequently, the *Q_unit_* can effectively guide the design of Flo‐TENG and aid in selecting optimal energy harvesting directions within the shell, as well as standardizing the assessment of Flo‐TENG.

## 2 Results and Discussion

### 2.1 Theoretical Analysis of Structural Quality Factor

**Figure 1:**
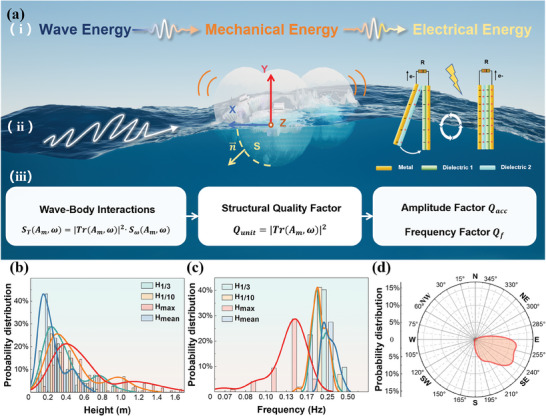
Illustration of Energy Transfer in Flo‐TENG and Statistical Graphs of Ocean Wave Characteristics. a) Illustration of energy transfer during wave‐body interaction. b) Histogram and fitted distribution curve of wave height probability density. c) Histogram and fitted distribution curve of wave frequency probability density. d) Radar plot of wave direction probability distribution. X‐axis: Direction of wave incidence on the shell; Y‐axis: Perpendicular direction within the same horizontal plane; Z‐axis: Upward vertical direction. H_1/3_, H_1/10_, H_max_, and H_mean_ correspond to significant, one‐tenth significant, maximum, and mean wave height, respectively (Note S1, Supporting Information).

Generally, a Flo‐TENG consists mainly of a shell, internal inertia units, and triboelectric layers between them. In the ocean, the energy transfer process can be illustrated in **Figure** **1**a(i‐ii). The Flo‐TENG moves under the action of water waves, converting mechanical energy from the wave into mechanical energy of the Flo‐TENG. As a result of this movement, inertia causes separation or sliding of the triboelectric layer within the internal TENG unit. This leads to alterations in polarization fields of dielectric materials and generates displacement currents that establish a closed‐loop circuit through external circuitry, facilitating the conversion of mechanical energy from the Flo‐TENG into electrical energy. Since ocean waves are random waves, the above energy transfer process should be discussed from a stochastic point of view. In addition, the inherent stochasticity of wave trajectories gives rise to random force vectors acting on the Flo‐TENG shell. This leads to diverse motions of the Flo‐TENG, resulting in varying electrical outputs for TENG units oriented in different directions. Therefore, comprehending the stochastic interaction between ocean waves and Flo‐TENG is pivotal in determining the efficacy of wave energy harvesting in the intended direction by Flo‐TENG (Note S2, Figures S1 and S2, Supporting Information).

Based on the ocean wave model proposed by Longuet‐ Higgins,^[^ ^44^, ^45^ ^]^ the wavefront at a fixed point is comprised of multiple component waves, which can be regarded as a linear superposition of waves with varying amplitudes (*A_m_*), frequencies (*f*), and phases (*p*). The total energy of an ocean wave is derived from the collective contribution of all its constituent waves. The distribution of ocean wave energy relative to the frequency of the component waves is called the wave spectrum. We designate the circular frequency of the constituent waves forming the actual ocean waves as *ω* and the energy spectrum denoted as *S*_ω_(*A_m_*,ω). With the excitation of random waves, the energy spectrum of the Flo‐TENG is denoted as *S_T_* (*A_m_*,ω). *S*_ω_(*A_m_*,ω) and *S_T_* (*A_m_*,ω) represent the energy densities of the wave energy and the energy absorbed and transformed by Flo‐TENG, respectively. According to the non‐linear transfer theory,^[^ ^46^, ^47^ ^]^ the energy transfer relationship of wave‐body interaction (Figure 1aiii) can be expressed by Equation (1)^[^ ^48^ ^]^

(1) $$S_{T}\;\left(A_{m},\omega \right)={\left|T_{r}\left(A_{m},\omega \right)\right|}^{2}\;\cdot S_{\omega }\left(A_{m},\omega \right)\;$$

where, *T_r_* (*A_m_*,ω) represents the transfer function of wave‐body interaction, which encompasses various practical factors affecting the transfer process, such as complex fluid parameters, the intricate effects of wind on waves, and shape‐changing parameters. The expression of *T_r_* (*A_m_*,ω) in the ideal state can be calculated by the modified Morison's formula (Note S3 and Figure S3, Supporting Information), but *T_r_* (*A_m_*,ω) in the real application is an extremely complex function, here we consider it as a whole to measure the efficiency of the energy conversion from waves to Flo‐TENG. Mathematically, the transfer function shows that the essence of the transfer process is the modulation of the energy distribution from the wave height spectrum to the acceleration spectrum of the device by the shell. Thus, the complex stochastic interaction process and energy transfer relationship can be analyzed through the transfer function *T_r_* (*A_m_*,ω). The square of the modulus of the transfer function *T_r_* (*A_m_*,ω) additionally represents the efficiency of energy transfer for different shell bodies in the wave‐body interaction. During the course of a wave, the average energy density in a period is directly proportional to both the square of its amplitude and frequency. Due to independent variations in the amplitude and frequency of natural ocean waves, we investigate the transfer function from two aspects: its response to the wave amplitude and its response to the wave frequency. Meanwhile, considering the wave directivity, we describe the transfer function in terms of different axes. Consequently, we have reformulated *T_r_* (*A_m_*,ω) as Equation (2).

(2) $$\begin{array}{c} T_{r}\;\left(A_{m},\omega \right)=T_{r_{1}}\;\left(A_{m}\right)\cdot T_{r_{2}}\;\left(\omega \right)=T_{r_{1}}\;\left(X_{A_{m}},Y_{A_{m}},Z_{A_{m}}\right)\cdot T_{r_{2}}\left(\omega \right) \end{array}$$

Here, *T*_*r*1_(*A_m_*) and *T*_*r*2_(ω) are the amplitude transfer function and the frequency transfer function, respectively. $X_{A_{m}},Y_{A_{m}},Z_{A_{m}}$ are the wave amplitude along X, Y, and Z axes. We hereby define the structural quality factor (*Q_unit_*) of a Flo‐TENG shell as given by Equation (3):

(3) $$Q_{unit}={\left|T_{r}\left(A_{m},\omega \right)\right|}^{2}\;=\frac{S_{T}\left(A_{m},\omega \right)}{S_{\omega }\left(A_{m},\omega \right)}\;\propto \;P_{absorption}=\frac{1}{n}\;\sum_{i\;=\;1}^{n}P_{i}$$

where *P_absorption_* denotes the overall average energy conversion efficiency of the Flo‐TENG, *n* is the number of degrees of freedom of the Flo‐TENG, and *P_i_* represents the energy conversion efficiency of the Flo‐TENG on each degree of freedom. Nevertheless, it is important to acknowledge that the maximum value of the energy conversion efficiency, as defined by this method, is not 100%. When the input wave excitation is sufficiently small, and the amplitude and frequency of the shell's oscillation are sufficiently large, the limit of this quality factor becomes infinite. Based on this, the structural quality factor *Q_unit_*, proposed for structures under random wave excitation, has a clear physical meaning and is effective.

To obtain statistically significant structural quality factors, further statistical descriptions of *S*_ω_(*A_m_*,ω) and *S_T_* (*A_m_*,ω) are required. This involves obtaining statistical features representing amplitude and frequency parameters to describe *S*_ω_(*A_m_*,ω) and *S_T_* (*A_m_*,ω) in Equation (1). In statistical analysis of random waves, significant wave height (*H*_1/3_) and significant wave period (*T*_1/3_) commonly employed to characterize the average properties of waves. The wave height is commonly regarded as twice the wave amplitude, while the circular frequency ω can be obtained by taking the reciprocal of the period and multiplying it by 2π. Within one given wave period, the significant acceleration amplitude (*a*_1/3_) of wave can be expressed as:

(4) $$a_{1/3}=\frac{2\pi^{2}H_{1/3}}{T_{1/3}^{2}}\;\propto H_{1/3}$$

With the wave‐body interaction, the Flo‐TENG produces stochastic acceleration signals, where the amplitude and frequency of acceleration represent the responses to the wave amplitude and wave frequency, respectively. Through statistical analysis, the transfer function can be further written as:

(5) $$\left|{T_{r}}_{1}\left(A_{m}\right)\right|\propto \left|\frac{a_{\mathrm{pro}}}{a_{1/3}}\right|$$

(6) $$\left|{T_{r}}_{2}\left(\omega \right)\right|\propto \left|\frac{\omega_{\mathrm{pro}}}{\omega_{1/3}}\right|$$

where, *a_pro_* reflects the average level of the acceleration amplitude of Flo‐TENG. ω_*pro*_ reflects the average level of the acceleration frequency of the Flo‐TENG. ω_1/3_ reflects the average level of the significant frequency of wave under one given wave period, obtained by taking the inverse of *T*_1/3_ and multiplying it by 2π.

Finally, we quantify the amplitude structure quality factor (*Q_acc_*) and the frequency structural quality factor (*Q_f_*) as follows:

(7) $$Q_{acc}=\frac{a_{pro}^{2}}{a_{1/3}^{2}}\;$$

(8) $$Q_{f}=\frac{\omega_{pro}^{2}}{\omega_{1/3}^{2}}\;$$

By embedding an IMU in the Flo‐TENG shell, the acceleration data of the device can be achieved. Besides obtaining the statistical features of acceleration amplitude and frequency, we can further calculate the maximum probable energy absorbed by Flo‐TENG in a significant wave period, called the most possible energy value (*E_mp_*). This value characterizes the relative efficiency of energy conversion at different degrees of freedom under the same wave conditions, expressed by Equation (9).

(9) $$E_{mp}=\frac{1}{2}\;ma_{pro}^{2}T_{pro}^{2}$$

where *T_pro_* represent the motion time of a specific shell under the probable acceleration frequency in a significant wave period, obtained by dividing 2π by ω_*pro*_. *m* denotes the mass of the floating object.

We can see that there is a positive correlation between *Q_unit_* and energy absorption efficiency. A larger *Q_unit_* indicates a higher energy absorption efficiency of Flo‐TENG, while a smaller *Q_unit_* suggests lower efficiency. This provides a fundamental foundation for evaluating the energy conversion capabilities of various Flo‐TENG shells and facilitates the optimization of their design. Based on above analysis, we conduct a series of experiments to explore the impact of various shape parameters on *Q_acc_* and *Q_f_* in ocean.

## 3 Statistical Properties of Natural Ocean Waves

First, we analyzed the statistical characteristics of waves in the experimental sea area. We conducted measurements using a wave gauge to record the wave height, frequency, period, and direction of waves in the experimental sea area for 13 days, with time intervals of 30 min. The probability distribution histograms and Kernel smoothing fit curves (Figure 1b,c) for wave height and frequency and the radar chart depicting wave arrival directions (Figure 1d) were obtained through calculation and statistical analysis of the collected data. Based on the observation results, it was found that in the experimental sea area, the most probable value of the significant wave height, one‐tenth significant wave height, maximum wave height, and mean wave height were 0.15, 0.24, 0.31, and 0.41 m, respectively. Simultaneously, secondary peaks were observed at 0.46, 0.76, 0.97, and 1.18 m, as depicted in Figure 1b. Apart from H_max_, the most probable frequency for waves of different heights was ≈0.2 Hz, with a secondary peak frequency observed at 0.23 Hz. The difference between the two peak frequencies is minimal, as shown in Figure 1c. Additionally, Figure 1d indicates wave directions predominantly within the range of 235° to 270° (angles measured counterclockwise from the north in a polar coordinate system). The observational results indicate that the waves in the experimental sea area exhibit a bimodal distribution characteristic formed by the combined action of typical wind and swell waves. The waves in the experimental sea area are characterized by a narrow spectrum and randomness, with a certain degree of stability in wave direction, typical of shallow‐water waves. This also implies that TENGs operating in the natural ocean will continue to be subjected to wave excitation in the direction of wave propagation.

## 4 The Influence of Different Bow Shapes on Q_unit_

**Figure 2:**
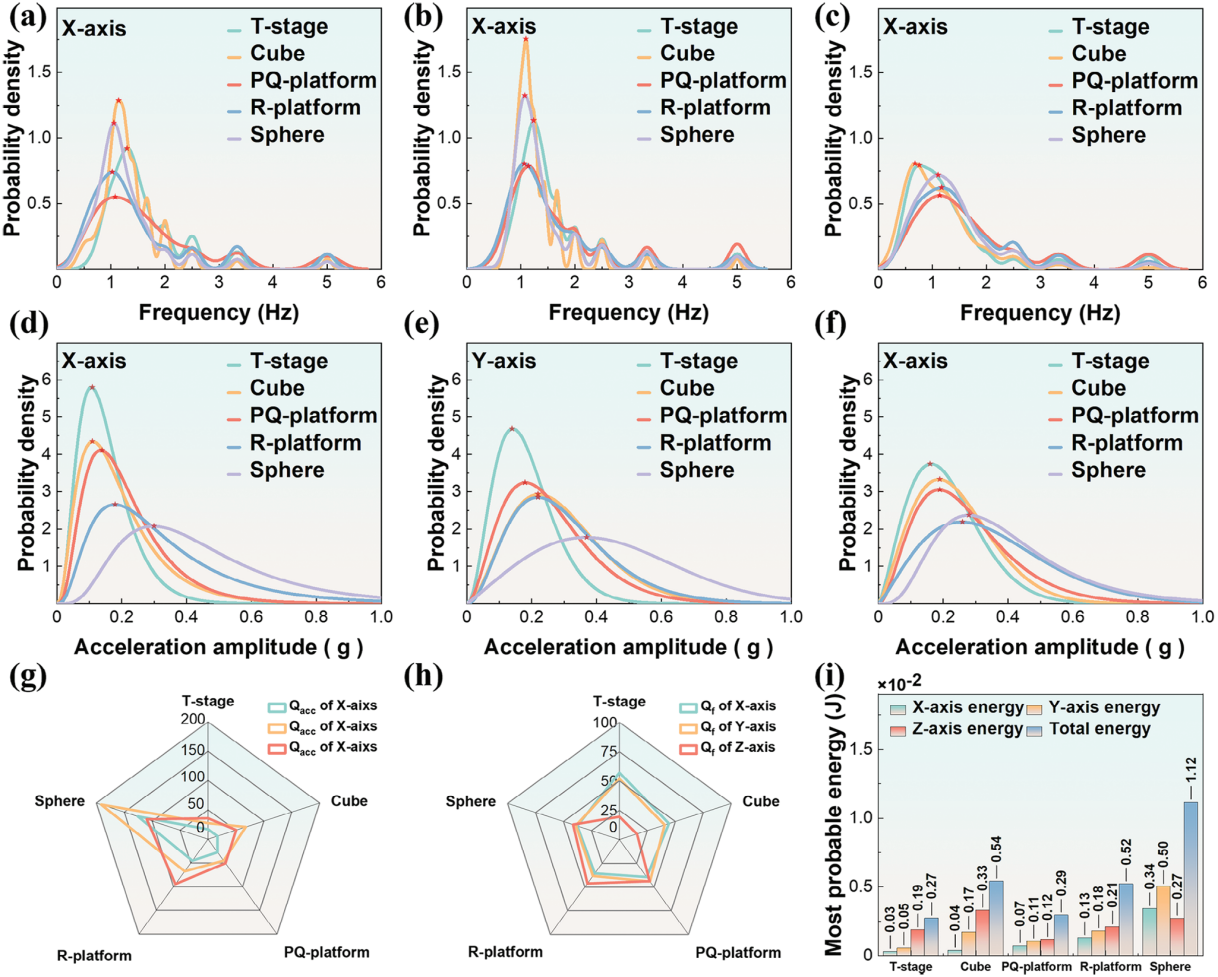
Wave‐Body Interaction Characteristics under Different Bow Shapes. a–c) Probability density histograms and curves of shell frequency along the X, Y, and Z axes for different bow shapes. d–f) Probability density histograms of shell motion amplitude along the X, Y, and Z axes for different bow shapes. g, h) Radar chart of shell amplitude structural quality factors (Q_acc_ and Q_f_) for different bow shapes. i) Most probable energy values for each shell axis and overall for different bow shapes. Red pentagrams indicate peak values.

In order to explore the effects of different bow shapes on structural quality factors and their physical mechanisms under natural wave action, we selected the surface S of the shell in the X‐axis direction as the wave‐body interaction surface in the experiments, as shown in Figure 1a. Five different shaped shells were chosen for study: Trapezoid stage, cube, positive quadrangular platform, round platform, and sphere, all with a diameter of 10 cm. The shells were designed following the principles of equal bow surface dimensions (Figures S4–S8, Supporting Information), equal center of gravity position (Figure S9 and Note S4, Supporting Information), and equal mass (m = 0.085 kg). The IMU unit (Note S5, Figure S10, and Table S1, Supporting Information) was embedded into devices of different shapes, with the X‐axis serving as the bow direction, the Y‐axis perpendicular to the bow direction in the same horizontal plane, and the Z‐axis oriented vertically upward. This setup enabled the measurement of the raw acceleration data of Flo‐TENG under the wave‐body interaction effect in natural ocean waves. Each device was tested for 30 min per group, repeated in 3 groups. Through the testing, we obtained raw acceleration data for five different axes. By analyzing and fitting the raw acceleration data using the maximum entropy method (Figure S11, Notes S6, and S7, Supporting Information), probability density curves of acceleration frequency along different axes were obtained (**Figure** **2**a–c), as well as probability density curves of acceleration amplitude (Figure 2d–f). Based on the most probable value from the probability density plots, radar charts for *Q_acc_* along different axes (Figure 2g), a *Q_f_* radar chart (Figure 2h), and a histogram for *E_mp_* (Figure 2i) were further calculated and plotted.

Under the excitation of ocean waves, the frequency probability density curve of acceleration amplitude reflects the frequency characteristics of the shell's motion. As shown in Figure 2a,b, on the X and Y axes, the most probable value of the five shell motion frequencies is concentrated ≈1 Hz. The number of most probable frequency values and high probability component peaks decreases in the order of Cube, Sphere, Trapezoid stage, Round platform, and Positive quadrangular platform. Combined with Figure 2c, it indicates that compared to the X and Y axes directions, the probability density of shell motion frequencies in the Z‐axis direction has a broader distribution range, lower probability of a single frequency occurrence, and fewer peaks of high‐frequency components.

Figure 2d–f respectively illustrate the probability density distribution of shell motion acceleration amplitudes along the X, Y, and Z axes. It can be observed from the figures that, in each direction, the probability density distribution of axial acceleration amplitudes approximately follows a Rayleigh or Weibull distribution, with only one peak value. The most probable value of acceleration amplitudes along each axis decreases in the order of sphere, round platform, positive quadrangular platform, cube, and trapezoid stage.

Figure 2g illustrates the *Q_acc_* values of shells with different bow shapes. The sphere shell exhibits a significant advantage in *Q_acc_* values along each axis, while the trapezoid stage shell shows a notable disadvantage across all axes. Interestingly, the cube and sphere shells exhibit higher *Q_acc_* values in the Y‐axis direction than other axes. Regarding the performance in *Q_f_*, the trapezoid stage and cube shell show lower frequency structural quality factors on the Z‐axis. In contrast, the remaining shells exhibit no significant differences in *Q_f_* values across axes. As shown in Figure 2i, the spherical shape exhibits the highest total most probable energy absorption value, with the most possible energy levels in each axis following a similar trend as the variation in *Q_acc_*. Compared to other shapes, the energy absorption levels of spherical shapes in each axis of the spherical shape are relatively high. The cube and the positive quadrangular platform exhibit similar total most probable energy absorption values, but the contribution of each axis to the total most likely energy differs between them. In the case of the cube, the energy absorbed along the Y and Z axes constitutes the primary contribution to the most probable energy. On the other hand, the total most likely energy in the positive quadrangular platform is contributed equally by all three axes.

It was indicated that centimeter‐scale Flo‐TENG shells, triggered by natural ocean waves, exhibit motion frequency responses that exceed the significant wave frequency (F_1/3_ = 0.2 Hz) and possess multiple high‐frequency components. On the one hand, the high‐frequency components are related to the intrinsic oscillations of the Flo‐TENG device. When subjected to wave forces, the equilibrium state of the device is disrupted, causing the center of gravity to deviate from its balanced position. Under gravity, the device will return to its equilibrium state. However, during the initial oscillation process, it still possesses kinetic energy even after the device returns to its balanced position. This results in the device deviating from the equilibrium position again under the inertia until the kinetic energy is depleted. Therefore, under the influence of a single wave action, the shell undergoes multiple oscillations, resulting in the emergence of the main high‐frequency components observed in Figure 2a–c. On the other hand, within a single wave cycle, the wave‐body interaction process of the Flo‐TENG shell is influenced by the stable waves during the mature phase and the low‐amplitude, high‐frequency fragmented waves generated after the wave breaks. At the same time, the lower presence of high‐frequency components in the Z‐axis indicates that the selected shell shapes in the experiment are more susceptible to the influence of fragmented waves on the X and Y axes while being less affected on the Z‐axis.

From the probability density distribution of acceleration along each axis shown in Figure 2d–f, it can be observed that smoother bow surfaces tend to exhibit higher peak acceleration values. This is because asymmetrical and non‐smooth bow surfaces tend to split the waves upon encountering wave impact. During a complete wave impact process, a stable and slow‐moving small wave accumulation area with a specific height will form within a certain distance from the bow and side surfaces of the shell. This area alters the direction and velocity of water flow around the shell, thereby dispersing the impact of water waves on the shell and reducing the force exerted on it, resulting in lower acceleration of the shell under wave action. We substantiated this conclusion through computational fluid dynamics (CFD) simulations (Figure S12 and Note S8, Supporting Information).

From the perspective of the most probable energy absorbed by each shell shape, it can be observed that the sphere, round platform, and positive quadrangular platform demonstrate higher energy absorption capabilities across all three axes. Conversely, the trapezoid stage and cube exhibit their energy absorption primarily along the Z‐axis. This suggests that asymmetry, lack of inclination, or absence of curvature on the bow surface will result in a distinct disadvantage in energy absorption capacity for the shell along the XOY plane. However, energy absorption capacity has a particular advantage along the Z‐axis.

The experimental results demonstrate that shells of different shapes exhibit varying degrees of compatibility with wave energy absorption efficiency and the conversion capabilities of acceleration amplitude and frequency along various axes. This indicates that the energy absorption capacity is directional. In future Flo‐TENG designs, it is imperative to consider further the compatibility between the operating mode of TENG units and the wave direction, as well as the influence of wave flow around the device. Based on the working characteristics of TENG power generation units, it is essential to design encapsulating shells that better match both the TENG power generation units and wave energy absorption.

## 5 The Influence of Different Wavefront Curvatures on Q_unit_

**Figure 3:**
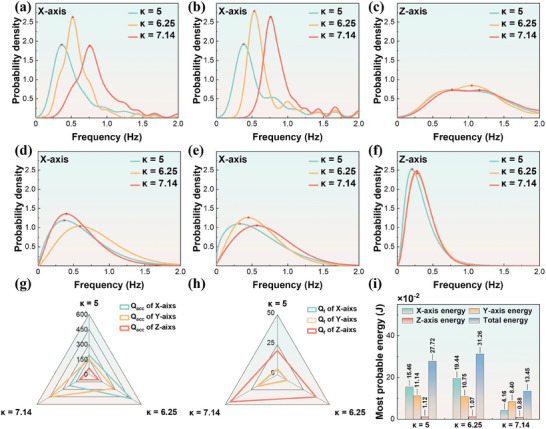
Wave‐Body Interaction Characteristics under Different Bow Curvatures. a–c) Probability density histograms and curves of shell frequency along the X, Y, and Z axes for different bow curvatures. d–f) Probability density histograms of shell motion amplitude along the X, Y, and Z axes for different bow curvatures. g, h) Radar chart of shell amplitude structural quality factors (Q_acc_ and Q_f_) for different bow curvatures. i) Most probable energy values for each shell axis and overall for different bow curvatures. Red pentagrams indicate peak values.

To further investigate the effect of bow shape smoothness, i.e., the curvature parameter, on the energy absorption efficiency of Flo‐TENG, we conducted experiments using three spherical devices with curvatures of κ = 5, κ = 6.25, and κ = 7.14, respectively. Under the conditions of an immersion depth of 3 cm, equal mass (m = 0.3 kg), and equal center of gravity position (one‐third of the height), the experiments were carried out using the same method. **Figure** **3**a,b illustrate that as the curvature κ increases, the most probable frequency values of the device increase along the X and Y axes. However, on the Z‐axis, the frequency response of different curvature devices varies into a broader range, with minimal impact from the curvature variation, as shown in Figure 3c. Figure 3d shows the probability density distribution curves of the acceleration amplitude along the X‐axis for different curvature devices. In the X‐axis direction, the most probable value of the acceleration amplitude exhibits a trend of first increasing and then decreasing with the increase of curvature κ. Conversely, in the Y‐axis direction, it increases with the rise in curvature κ, as depicted in Figure 3e. On the Z‐axis, the variation of curvature κ has a limited impact on the most probable value of the acceleration amplitude (Figure 3f). Figure 3g demonstrates that the variation of *Q_acc_* values follows a similar trend to the changes in the most probable acceleration amplitude shown in Figure 3d–f. The *Q_f_* values increase with the increase of curvature κ, with the Z‐axis exhibiting larger *Q_f_* values compared to the X and Y axes, as shown in Figure 3h. Figure 3i illustrates that the total most probable energy absorbed by the shell increases initially with the curvature parameter κ and then decreases. The most probable energy values along the Y and Z axes of the shell remain almost unchanged across different curvatures.

It was shown that the shell motion frequency increases with the curvature parameter κ within a specific curvature range on the X and Y axes. This suggests that within this range, increasing the curvature of the shell allows for better alignment with the wave action in the XOY plane, enabling the shell to achieve higher motion frequencies under the same wave excitation over a certain period. The probability density distribution of acceleration amplitudes exhibits distinct characteristics on the X and Y axes. This suggests that shells with different curvatures form distinct accumulation patterns and flow velocity distributions of small wave accumulation areas along the X and Y axes, influencing the acceleration variations. The variations in *Q_acc_*, *Q_f_*, and *E_mp_* suggest that changes in curvature have a minimal impact on *Q_f_*. Furthermore, curvature primarily affects *E_mp_* by altering the oscillation amplitude along the X‐axis of the shell, thereby influencing the total energy absorption. This further illustrates the matching relationship between curvature and wave forces in the bow direction. When curvature aligns with wave forces, larger oscillation amplitudes are generated, whereas smaller amplitudes are produced when mismatched.

## 6 The Influence of Different Inclination Angles on Q_unit_

**Figure 4:**
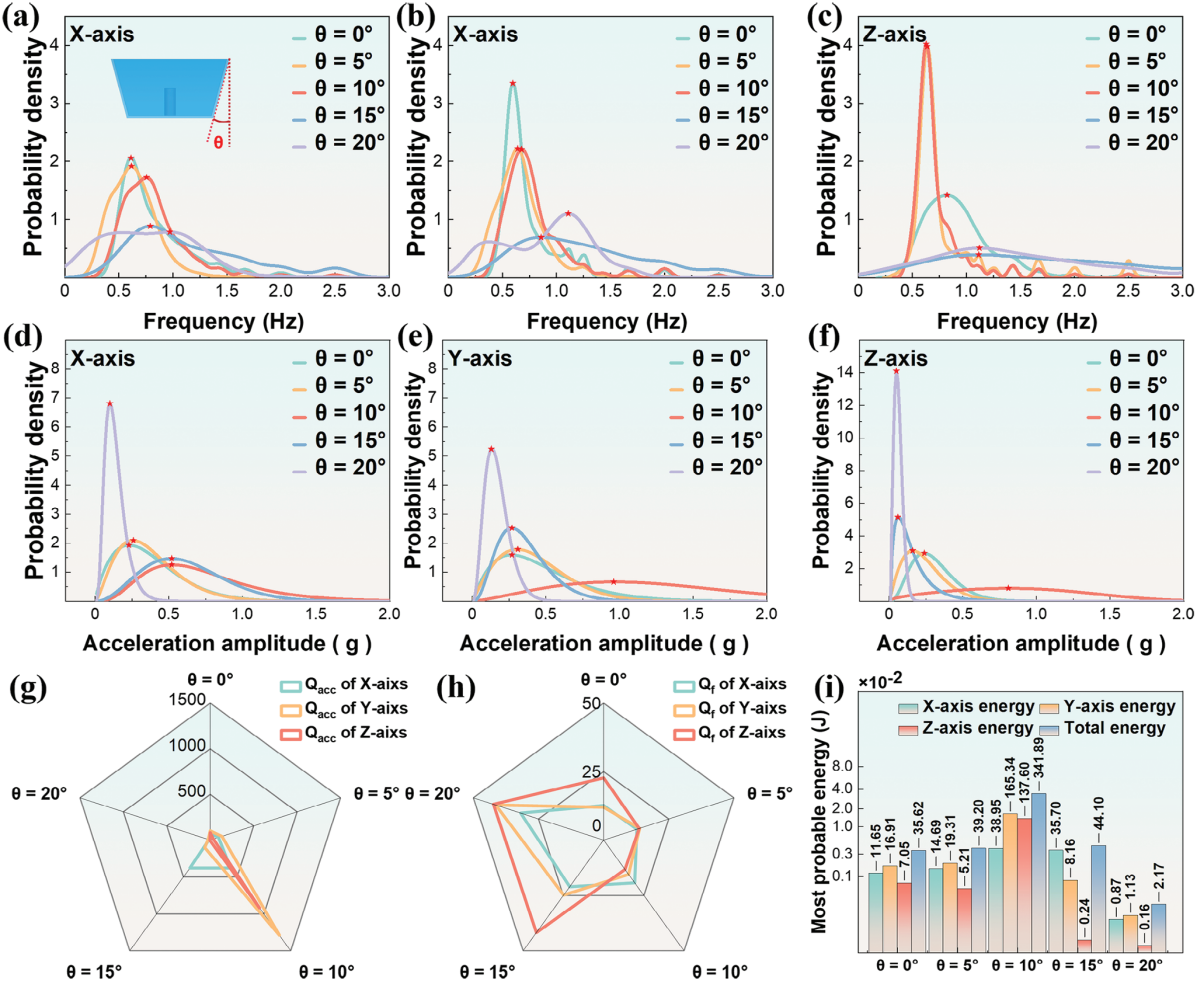
Wave‐Body Interaction Characteristics under Different Bow Tilt Angles (θ). a–c) Probability density histograms and curves of shell frequency along the X, Y, and Z axes for different bow tilt angles. d–f) Probability density histograms of shell motion amplitude along the X, Y, and Z axes for different bow tilt angles. g, h) Radar chart of shell amplitude structural quality factors (Q_acc_ and Q_f_) for different bow tilt angles. i) Most probable energy.

To investigate whether the inclined angle of the bow affects the compelling force of waves on the bow, we conducted experiments using five different tilt angles (θ = 0°, 5°, 10°, 15°, 20°) of a positive quadrangular platform (20 × 20 × 20 cm). The experiments were carried out under the same conditions of immersion depth (d = 10 cm), mooring weight (1 kg mooring weight block), mass (m = 0.65 kg), and center of gravity position (one‐third of the height) using the same methodology in natural sea wave conditions. **Figure** **4**a–c respectively depict the probability density distributions of the device's motion frequencies on the X‐axis, Y‐axis, and Z‐axis under different bow inclinations. At θ = 0°, 5°, and 10°, single peaks are observed on each axis, with the most probable frequency values distributed ≈0.5 Hz. The frequency distribution curves broaden as θ = 15° and 20°, increasing the most probable frequency values. On each axis, the most probable value of acceleration amplitude initially increases and then decreases with the increase in the inclination angle θ of the bow surface, indicating an optimal θ that maximizes the most probable acceleration amplitude. At θ = 10°, the maximum most probable acceleration amplitudes appeared on the X, Y, and Z axes, reaching 0.52, 0.96, and 0.81 g (g = 9.8 m /s^2^) respectively, as shown in Figure 4d–f.

Figure 4g illustrates the impact of the bow surface inclination angle on *Q_acc_*. At θ = 10°, a pronounced advantage is observed on the Y and Z axes, while on the X axis, the values of *Q_acc_* at θ =  10° and θ = 15° are similar and significantly greater than at other angles.

Figure 4h presents the values of *Q_f_* at different inclination angles of the bow surface. As the bow surface inclination angle increases, the *Q_f_* values on the X and Y axes remain unchanged. In contrast, on the Z axis, *Q_f_* increases initially before decreasing, reaching its maximum at θ = 15°. From Figure 4i, it can be observed that the total most probable energy increases initially and then decreases with the increasing inclination angle of the bow surface, reaching its maximum value of 341.89 J at θ = 10°. When θ exceeds 10°, there is a sharp decrease in the most probable energy value along the Z‐axis, decreasing from 137.60 J at θ = 10°, –0.24 J at θ = 15° and 0.16 J at θ = 20°. Similar reductions to varying degrees are also observed along the X and Y axes.

It was found that the shell exhibits better energy absorption efficiency when θ = 10°, suggesting the existence of an optimal shell incident wave angle maximizing the effective force exerted by the waves on the shell. This angle closely aligns with the complement of the wavefront obliquity angle formed after the interaction of waves with the bow‐induced deceleration flow field. This suggests that the shell can absorb more wave energy when the direction of wave action aligns parallel to the average direction of the shell's incident wave face (Figure 1a). Furthermore, a single curvature κ can be regarded as an aggregation of incident wave faces with various inclination angles, exhibiting better adaptability to the random process of natural ocean waves' directions and facilitating efficient conversion of the wave energy from multiple waveforms. This aligns with the observed trend in Figure 2i.

### 6.1 The Influence of Different Immersion Ratio on Q_unit_

To investigate the impact of different wave action positions on Flo‐TENG motion, we selected spherical devices with a diameter of 20 cm. Under conditions of constant curvature (κ = 5), constant mooring weight (1 kg mooring weight block), and continuous center of mass position (one‐third of the height), we controlled the immersion ratio by increasing the ballast weight (m = 0.2, 0.2625, 0.3375 kg). The experiments were conducted using the same methodology.

**Figure 5:**
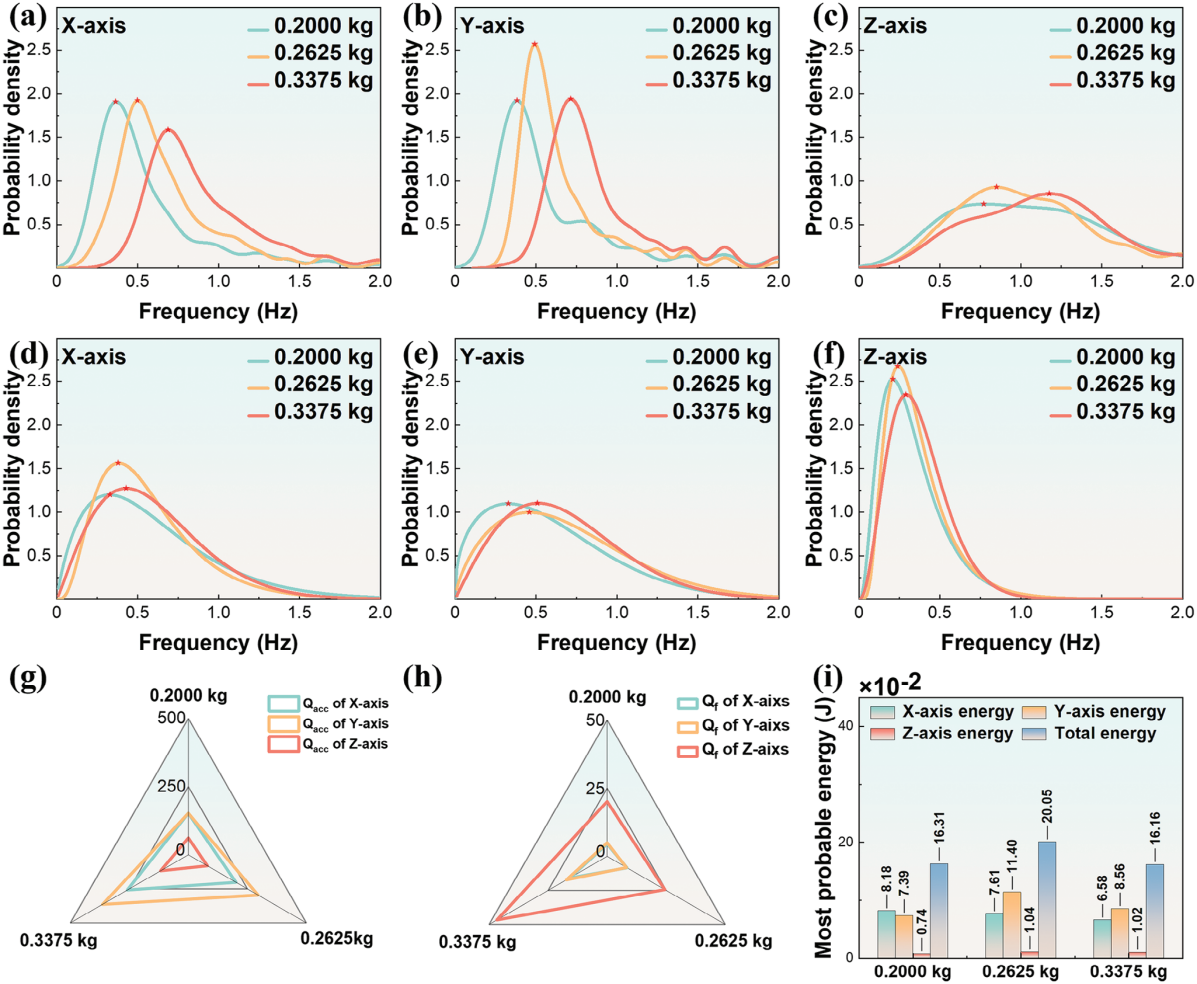
The Wave‐Body Interaction Characteristics under Different Immersion Ratios. a–c) Probability density histograms and curves of shell frequency along the X, Y, and Z axes for different immersion ratios. d–f) Probability density histograms of shell motion amplitude along the X, Y, and Z axes for different immersion ratios. g, h) Radar chart of shell amplitude structural quality factors (Q_acc_ and Q_f_) for different immersion ratios. i) The most probable energy values for each shell axis and different immersion ratios overall. Red pentagrams indicate peak values.

**Figure** **5**a,b depict the increase in the most probable frequency along the X, Y, and Z axes as the immersion ratio of the wave face increases. Notably, the Z‐axis exhibits a more comprehensive frequency distribution range (Figure 5c). In different axes, the most probable value of acceleration amplitude shows a slight increase with the increase in immersion ratio (Figure 5d–f). Figure 5g,h demonstrates that with the increase in immersion ratio, *Q_acc,_* and *Q_f_* show a slight increase in all axes. Adding mooring weights to the Flo‐TENG results in a significant decrease in energy absorption along the Z‐axis. With the increase in immersion ratio, the most probable energy on the Y and Z axes initially increases and then decreases. At the same time, on the X‐axis, there is a trend of decrease, albeit minor. The experimental results indicate that the variation in immersion ratio has a minor impact on the structural quality factor and energy absorption. The influence of the immersion ratio is primarily manifested in the energy absorption of the shell along the Y and Z axes.

### 6.2 Comparative Analysis of Laboratorial Water Waves and Natural Ocean Waves

**Figure 6:**
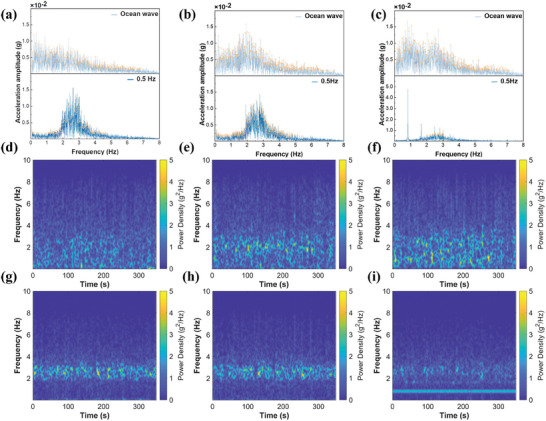
Real versus Laboratory Ocean Wave Characteristics: Amplitude‐Frequency and Energy Distribution. a) Frequency domain comparison of X‐axis acceleration signals for 0.5 Hz laboratory and natural ocean waves. b) Frequency domain comparison of Y‐axis acceleration signals for 0.5 Hz laboratory and natural ocean waves. c) Frequency domain comparison of Z‐axis acceleration signals for 0.5 Hz laboratory and natural ocean waves. d–f) Spectrograms of acceleration signals for natural ocean waves on the X, Y, and Z axes. g–i) Spectrograms of acceleration signals for 0.5 Hz laboratory waves on the X, Y, and Z axes.

Laboratory simulations often utilize wave makers or paddle systems to generate monochromatic waves to mimic natural oceanic conditions. However, waves generated through such methods exhibit significant disparities in characteristics compared to those naturally occurring in the open ocean. The disparities introduced by testing conditions make it challenging to accurately assess the performance of TENG devices in natural oceanic environments, rendering them impractical for use at sea. Therefore, investigating the similarities and differences between laboratory wave generation characteristics and natural ocean wave properties guides selecting laboratory wave parameters and devising wave generation methods. This is crucial for the practical implementation of TENG devices. Here, we conducted wave‐body interaction experiments under both natural ocean waves and laboratory wave generation conditions using a positive quadrangular platform shell (20 × 20 × 20 cm) with a tilt angle of θ = 10° and the center of gravity positioned at one‐third of the height. The laboratory wave generation involved a central frequency of 0.5 Hz and a power of 50W × 30 units, with the wave generator tilted at 5° counterclockwise from the horizontal plane. Each test lasted 30 min, and we repeated the experiments thrice. The acceleration frequency domain characteristics for each axis obtained through IMU testing are depicted in **Figure** **6**.

According to Figure 6a–c, the spectrum distribution of ocean waves is broad, primarily spanning between 0 and 5 Hz, which we refer to as the frequency bandwidth. In contrast, the laboratory‐simulated wave frequency bandwidth is narrow, as depicted in Figure 6a,b. Specifically, on the Z‐axis, the laboratory‐simulated waves exhibit only five significant components at frequencies of F = 0.84, 1.66, 2.48, 2.84, and 3.03 Hz, presenting sharp peaks with no apparent bandwidth, as shown in Figure 6c.

Figure 6d–f presents the spectrograms of the acceleration signals of the shell under the action of natural ocean waves in different axes. In spectrograms, higher energy appears in bright yellow, while lower energy occurs in blue. According to the results shown in the spectrograms, the energy distribution of natural ocean waves on all axes exhibits a uniform band‐like pattern that does not shift with changes in time and frequency.

Figure 6g,h depict the spectrograms of the acceleration signals of the shell in the X and Y axes under the influence of laboratory‐simulated 0.5 Hz waves. The energy distribution of laboratory‐simulated waves on the X and Y axes appears as a narrow band around F = 2.5 Hz, with a bright middle and dimmer upper and lower sides. On the Z axis, the energy distribution of laboratory‐simulated waves manifests as a bright line near F = 0.84 Hz and a narrow band above F = 1.66 Hz that becomes brighter before fading as the frequency increases.

These findings indicate that natural ocean waves exhibit more prosperous frequency components on all axes, with continuous frequency component distribution. Additionally, the energy distribution of natural ocean waves features a wide frequency bandwidth and remains stable without shifts in time or frequency. While exhibiting some frequency bandwidth on the X and Y axes, the laboratory‐simulated waves lack low‐frequency, high‐amplitude components. On the Z‐axis, the frequency components are singular, failing to replicate the nearly continuous frequency distribution observed in natural ocean waves. Multiple main components higher than the wave generation frequency appear on the Z‐axis, indicating that the wave tank's limited size contributes to numerous high‐frequency reflected wave components in laboratory waves. The laboratory‐generated waves exhibit stability akin to natural ocean waves, with their energy distribution remaining unaltered over time and frequency.

The experimental results show that the energy distributions of both laboratory wave‐making waves and real wave action are stable and do not shift with time and frequency. Compared with laboratory waves, the motion of real waves has more frequency components and a wider frequency distribution range. In addition, the amplitude of real waves is much smaller than that of laboratory waves. This is probably the main reason for the large difference in the output of the Flo‐TENG device between real waves and laboratory wave tests. In addition, we discuss the limitations and implications of the above data (Supplementary Note S9).

## 7 Conclusion

In summary, we propose a structural quality factor (*Q_unit_*) for Flo‐TENG shells to facilitate quantitative evaluations of their water wave energy conversion capabilities across diverse natural wave conditions. This offers essential guidance for designing high‐performance Flo‐TENGs and selecting optimal internal energy harvesting directions. Meanwhile, systematic experiments were conducted under natural ocean conditions and the influence rule of shell shape parameters on the *Q_unit_* is revealed. Significant findings were obtained by comparing the probability density distributions of acceleration amplitudes and frequencies, *Q_unit_*, and most probable energy values and conducting time‐domain analysis of ocean and laboratory wave acceleration signals. They include:

- Different shells exhibiting distinct axial characteristics in terms of quality factors.
- Spherical shells demonstrate superior energy absorption capabilities compared to other shell types.
- Cubic shells display better energy absorption capabilities along the Z‐axis.
- Higher energy absorption conversion efficiency of Flo‐TENG shells when the bow angle is approximately half the complementary angle of the wavefront angle.

It was observed that for centimeter‐to‐decimeter scale Flo‐TENGs their frequency components of acceleration amplitudes are nearly continuous distribution ranging from 0 to 5 Hz in natural ocean waves. In contrast, laboratory signals tended to cluster around a single frequency with a narrow bandwidth. And the amplitude of real waves is much smaller than that of laboratory waves. This is probably the main reason for the large difference in the output of the Flo‐TENG device between real waves and laboratory wave tests. We are also aware that the comparison of laboratory waves with real waves in the text is difficult to further standardize and systematize due to practical conditions, causing the resulting data to be difficult to quantitatively guide the reproduction of waves with wave characteristics in the laboratory. Furthermore, we can foresee that although *Q_unit_* is difficult to evaluate for all types of TENGs. But up to now, it is still considered an effective means of evaluating energy transfer between random excitations and Flo‐TENGs. In future research, the applicability and potential challenges of *Q_unit_* in real‐world scenarios will continue to be explored and addressed.

In conclusion, the findings presented in this study establish a solid foundation for the design of high‐performance Flo‐TENG, thereby significantly advancing the development and practical implementation of TENG‐based ocean kinetic energy harvesters.

## 8 Experimental Section

### Comparison of Laboratory and Natural Ocean Wave Experiments

In the laboratory setting, wave simulation was conducted in a giant wave pool (120 × 100 × 100 cm). Wave generation was facilitated by 30 wave pumps (Jiebao RW20), each with a power of 50 W, utilizing a fixed‐frequency square wave signal. The testing site selected for experiments under natural ocean conditions was the Beihai Sea area in China. Additionally, acceleration signals were collected using an inertial measurement unit (IMU) from Wit Motion, model BWT901BLECL5.0, with a sampling frequency of 20 Hz and a baud rate of 115 200. Each test session consists of continuous measurements over 30 min, repeated for three sets of trials.

### Fabrication Method of Floating Shell

All shells used in the experiments were constructed using UG NX12.0 to establish 3D models, from which parameters such as the centroid position were derived, facilitating the overall calculation after weighting. The wall thickness of each model was set to 2 mm, with an M6 screw fixing base in the middle of each model, allowing for the adjustment of the center of gravity height using nuts to control the weighting blocks. The spherical shell was made of acrylic material to observe the wave accumulation more effectively. The remaining shells were printed using R4600 epoxy resin. Before testing, a small amount of waterproof silicone (Kafuter 706) and waterproof tape (Newly born) were used for encapsulation.

### Data Processing Method

Data processing includes data acquisition, zero‐offset removal (Note S10, Supporting Information), outlier removal (Note S11, Supporting Information), filtering (Note S12, Supporting Information), maximum entropy fitting, and K‐S testing (Note S13 and Figure S14, Supporting Information). The raw acceleration data collected were represented by a_x_, a_y_, and a_z_, obtained through an IMU sensor. Due to potential installation and manufacturing imperfections in the IMU, zero‐offset removal was performed by subtracting the arithmetic mean of all data along a particular axis from the acceleration data along that axis to enhance the accuracy of the final calculations. Additionally, the Kalman filtering code written in the IMU cannot effectively filter out abnormal acceleration components caused by the dragging force of the mooring line on the shell and high‐frequency components induced by nonwave signals. In this study, the median absolute deviation method was employed to address these issues, taking advantage of the characteristics of large acceleration changes caused by dragging forces and short durations of action. Precisely, the median of the adjacent 20 data points in the signal was calculated, and acceleration points exceeding three times the median were replaced with the median value to filter out outliers. Moreover, a Butterworth low‐pass filter eliminates high‐frequency components above 5 Hz (Figure S15, Supporting Information Following data preprocessing, the three‐parameter method was employed to compute the fitting parameters of the maximum entropy distribution for the acceleration's maximum entropy distribution. Subsequently, a maximum entropy distribution fitting was conducted on the probability density function of amplitude, followed by a Kolmogorov‐Smirnov test (K‐S test) with a precision of 0.05 on the fitted probability density function. For the more random frequency characteristics, Gaussian kernel fitting was applied. The data above calculations and results serve as crucial references for the comparison of experimental outcomes.

## Conflict of Interest

The authors declare no conflict of interest.

## Author Contributions

D.G. conceived the project and designed the experiment part. D.G., J.L., and L.Z. reviewed papers about floating TENGs and IoTs. D.G., C.C., and S.L. fabricated the devices and completed the tests. D.G. and J.L. collaborated on the CFD calculations. S.H. and J.F. helped build the experimental setup and provided algorithmic support. D.G., L.W., G.L., and J.Z. wrote the manuscript. L. W. and G. L. supervised the project.

## Supporting information

Supporting Information

## Data Availability

The data that support the findings of this study are available from the corresponding author upon reasonable request.
