# Supplementary material for: Structural Quality Factor of Flo‐TENG under Stochastic Wave Excitation
Source: Adv Sci (Weinh). 2024 Aug 9;11(38):2405165. doi: 10.1002/advs.202405165 (PMC11481207; doi:10.1002/advs.202405165)
Supplement: Supplementary file 1 — Supporting Information [file ADVS-11-2405165-s001.docx]

**Supporting Information**

Structural Quality Factor of Flo-TENG under Stochastic Wave Excitation

*Dongxin Guo, Chunjin Chen, Jiawei Li, Lixia Zhai, Songying Li, Sheng He, Junrui Feng , Lingyu Wan*, Guanlin Liu*, and Junyi Zhai**

D. Guo, C. Chen, J. Li, L. Zhai, S. Li, S. He, J. Feng, Prof. L. Wan, Prof. G. Liu

Center on Nanoenergy Research, Institute of Science and Technology for Carbon Peak & Neutrality, State Key Laboratory of Featured Metal Materials and Life-cycle Safety for Composite Structures, School of Physical Science & Technology, Guangxi University, Nanning 530004, China

E-mail: lyw2017@gxu.edu.cn, guanlinliu@gxu.edu.cn

Prof. J. Zhai

CAS Center for Excellence in Nanoscience Beijing Key Laboratory of Micro-nano Energy and Sensor, Beijing Institute of Nanoenergy and Nanosystems Chinese Academy of Sciences, Beijing 101400, School of Nanoscience and Technology University of Chinese Academy of Sciences, Beijing 100049, China

E-mail: jyzhai@binn.cas.cn


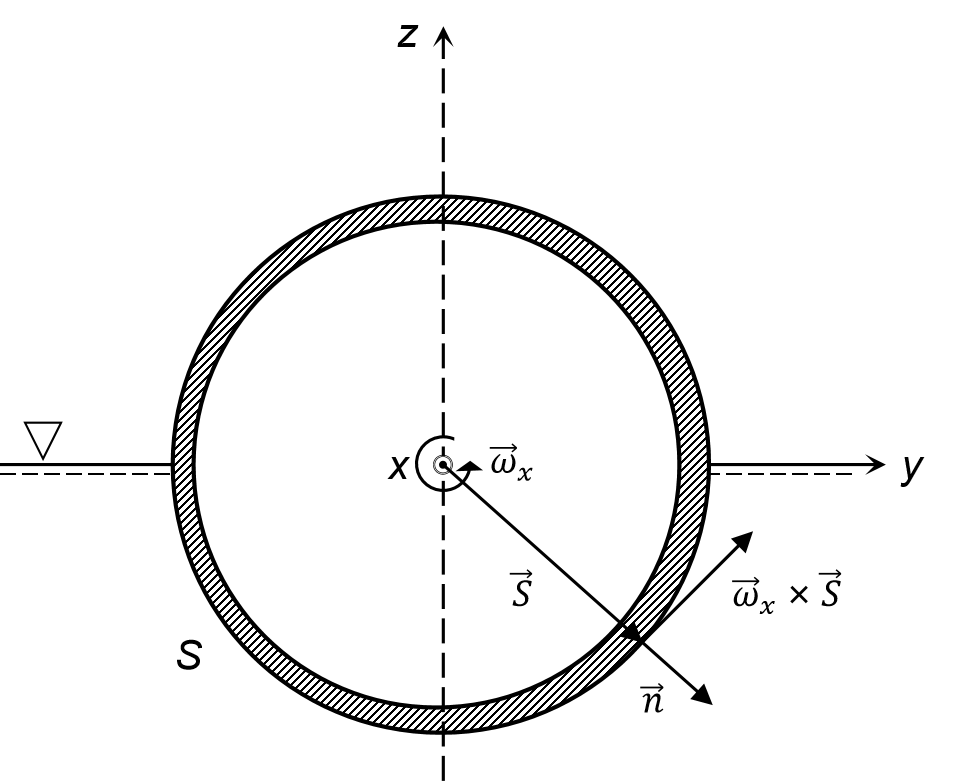


**Figure S1.** Floating body oscillating in water. Vector gives the position of a point on the wet surface (or bow face) S, where the unit normal is$\vec{n}$. It is the angular velocity vector corresponding to rotation about the x-axis.


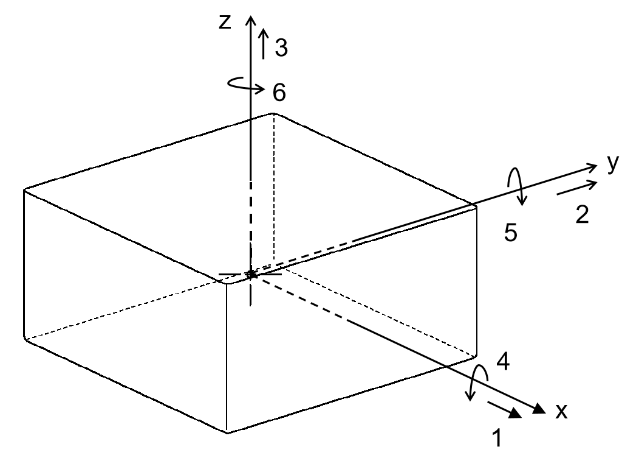


**Figure S2.** Floating body six-axis motion schematic. A floating body has six modes of motion: surge(1),sway(2),heave(3),roll(4),pitch(5) and yaw(6).


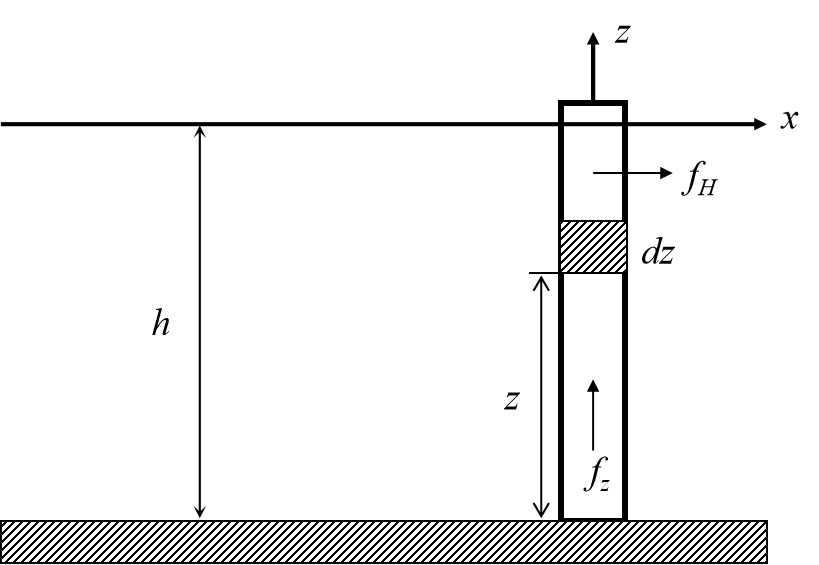


**Figure S3.** Schematic diagram of floating body model.


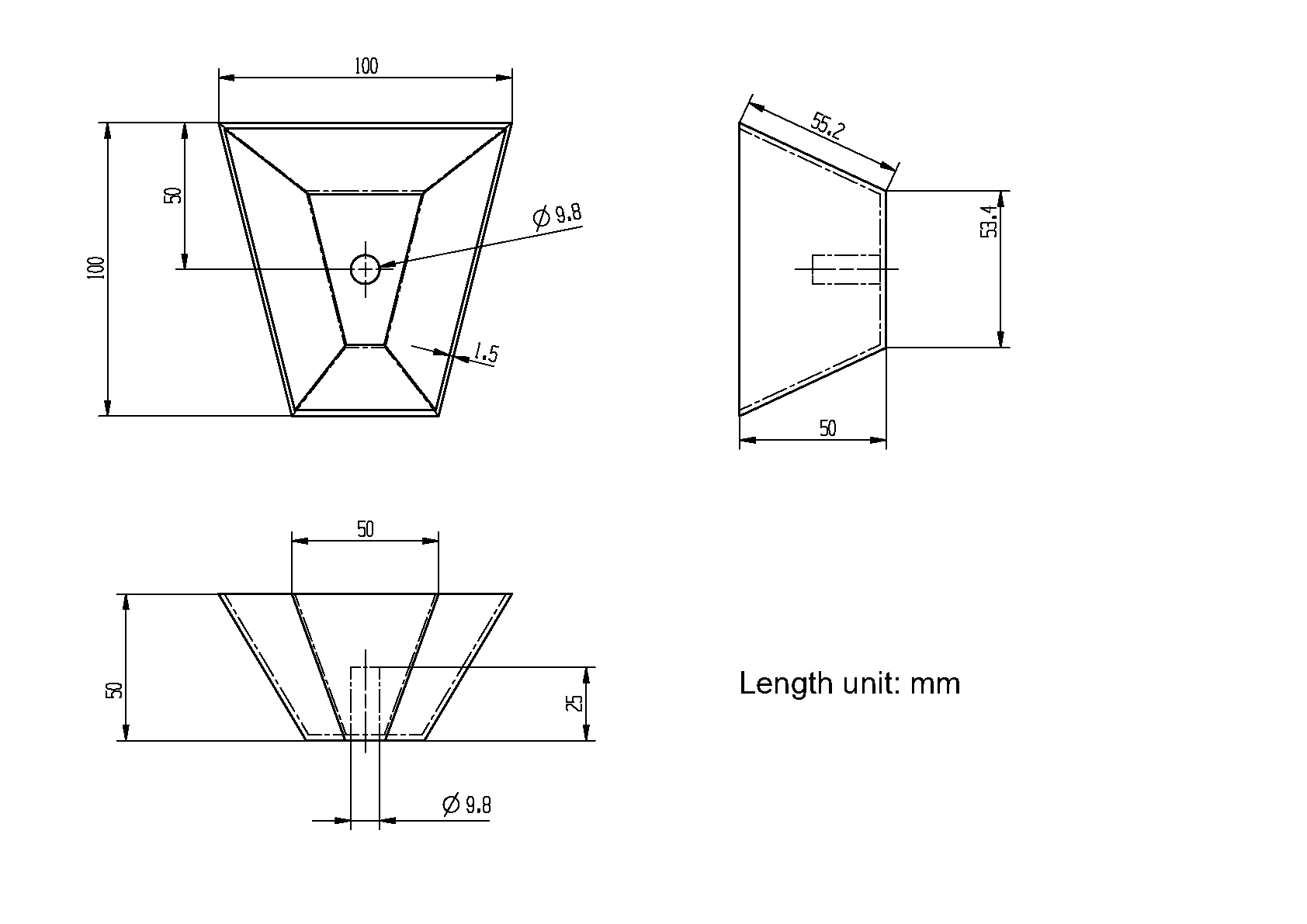


**Figure S4.** Schematic dimensions of the trapezoid stage.


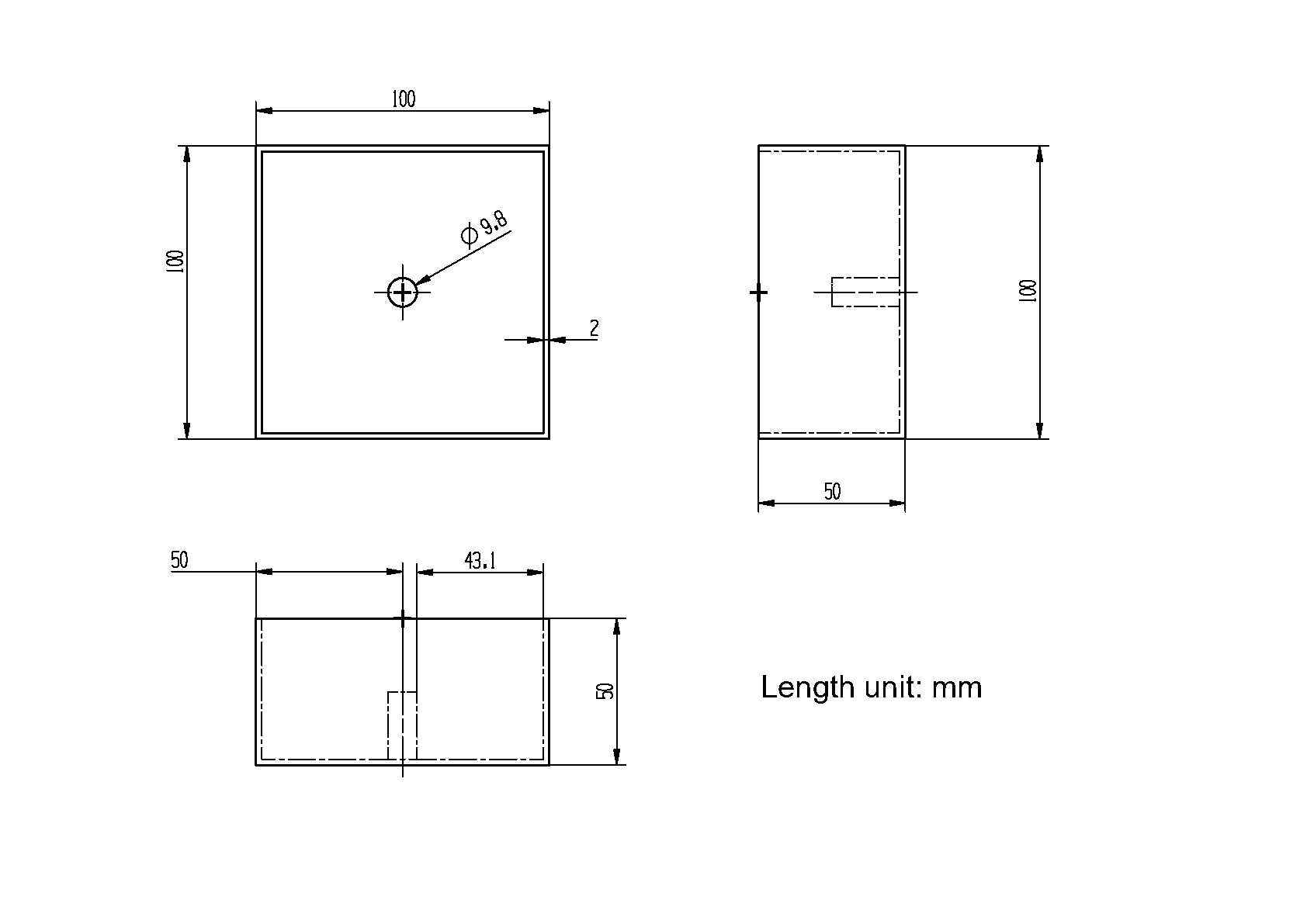


**Figure S5.** Schematic dimensions of the cube.


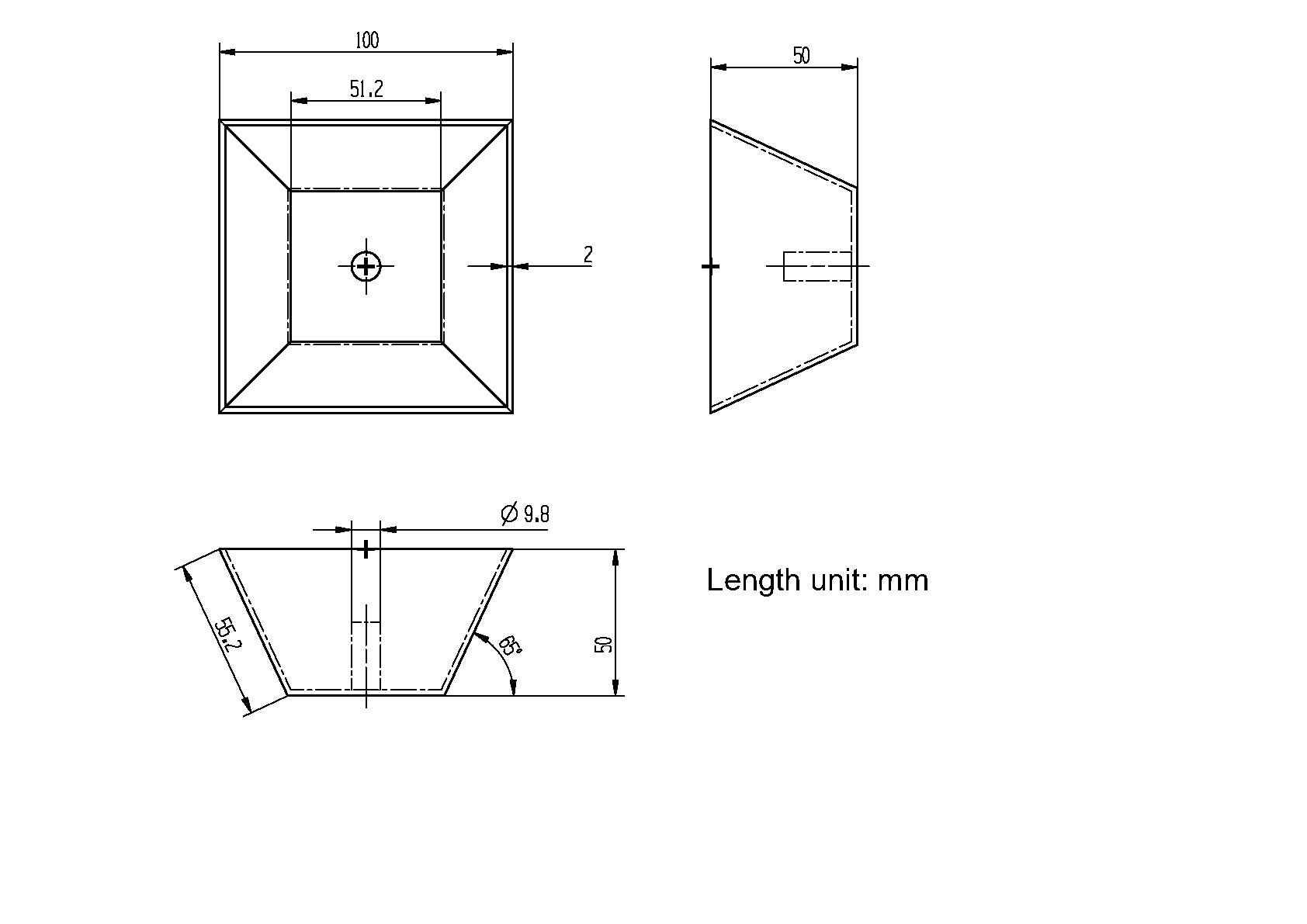


**Figure S6.** Schematic dimensions of the positive quadrangular platform.


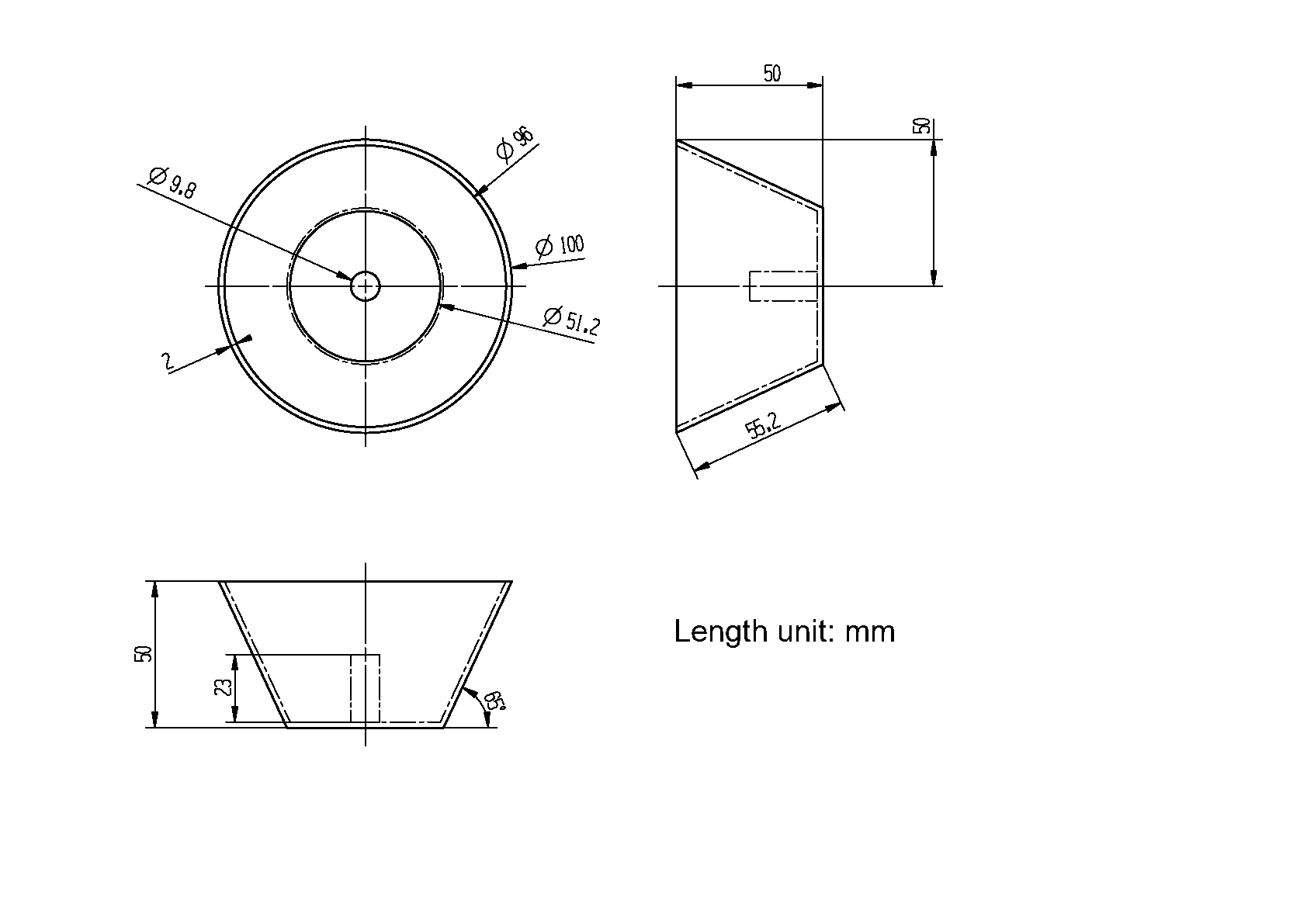


**Figure S7.** Schematic dimensions of the round platform.


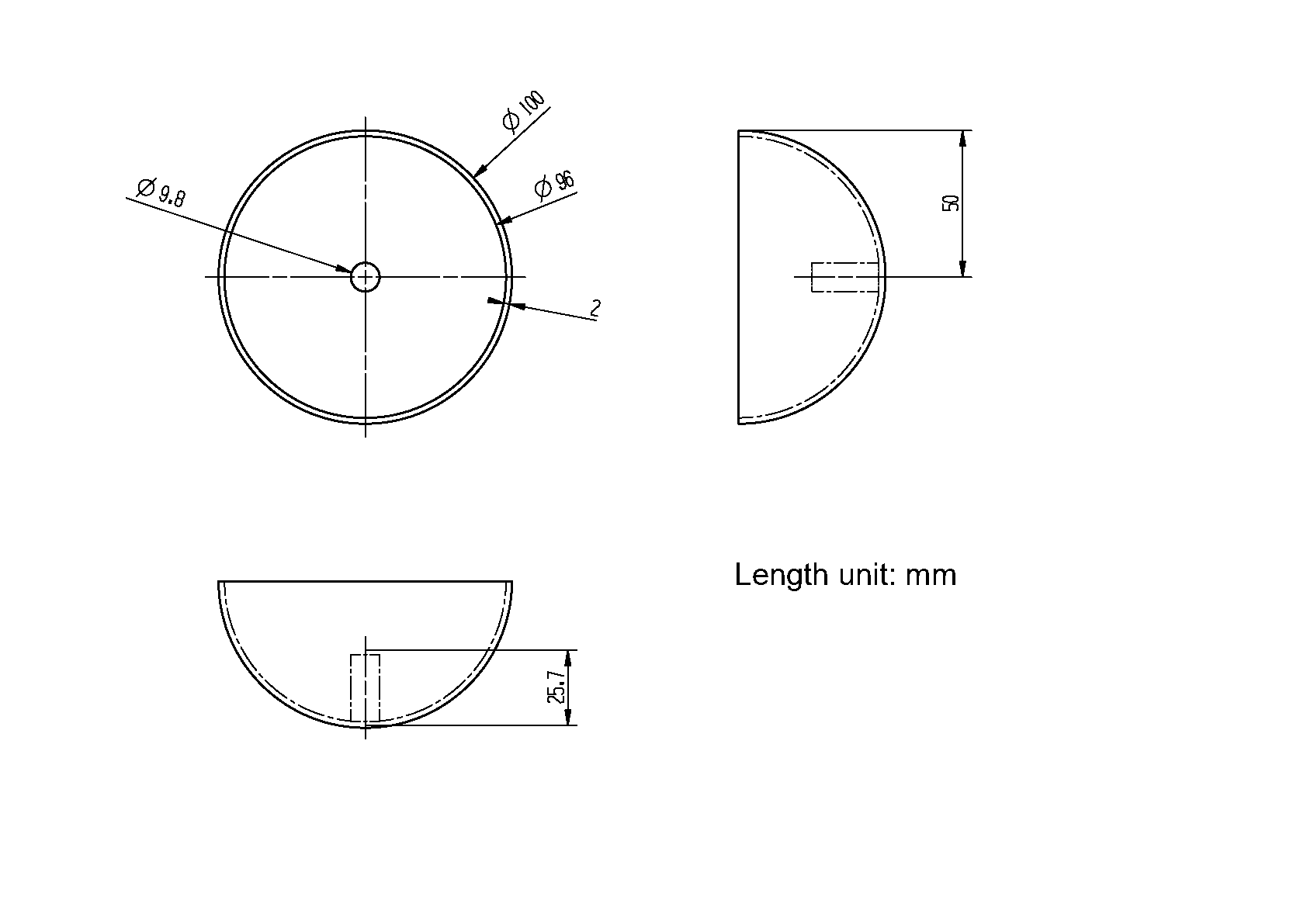


**Figure S8.** Schematic dimensions of the sphere.

**
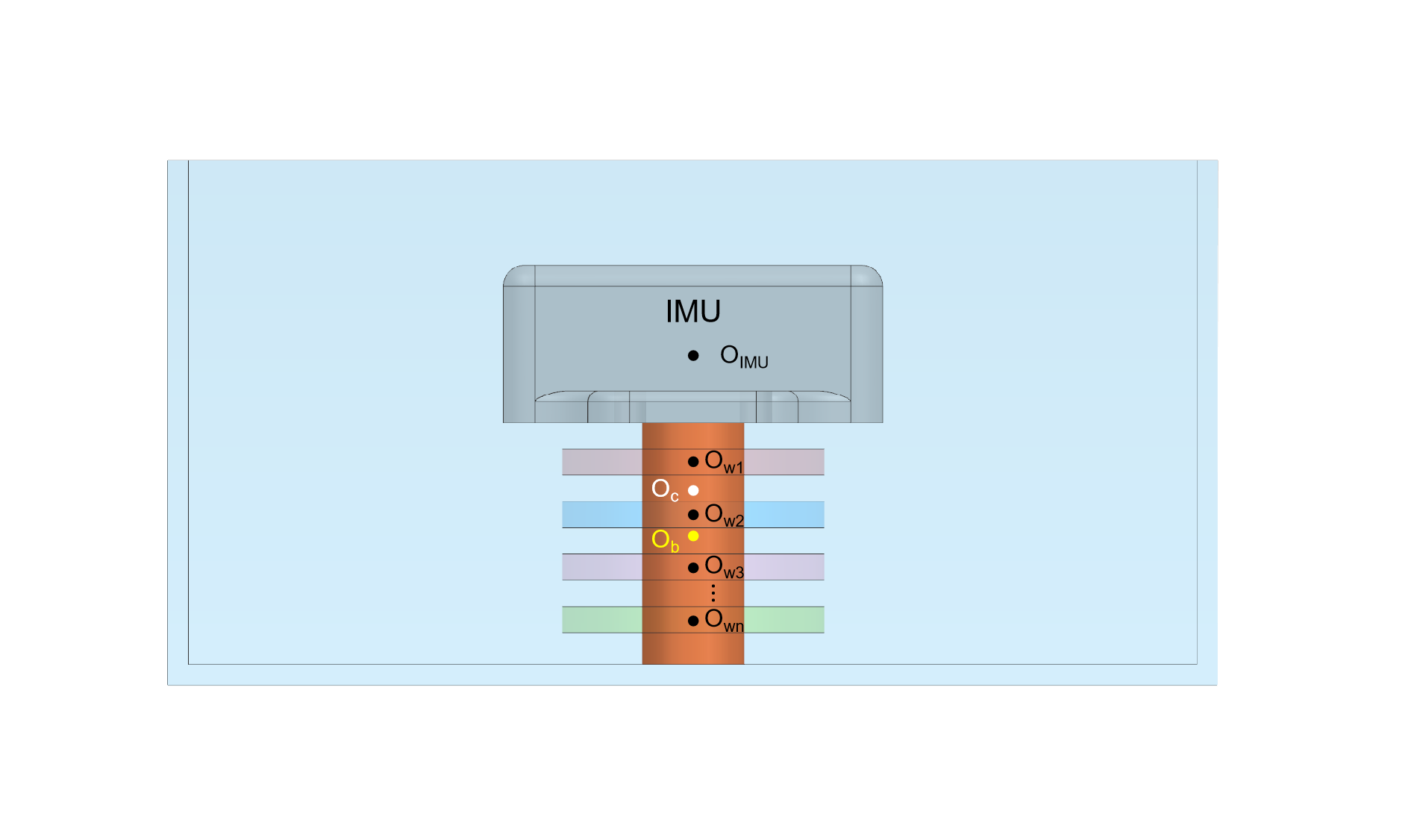
**

**Figure S9.** Schematic diagram of the center of gravity of the test equipment. The O_IMU_ represents the position of the center of gravity of the IMU. The center of gravity positions of the measured object and the floating body are denoted as O_b_ and O_c_, respectively. Furthermore, the center of gravity positions of the counterweight blocks are indicated as O_w1_, O_w2_, O_w3_, and so on, up to O_wn_.


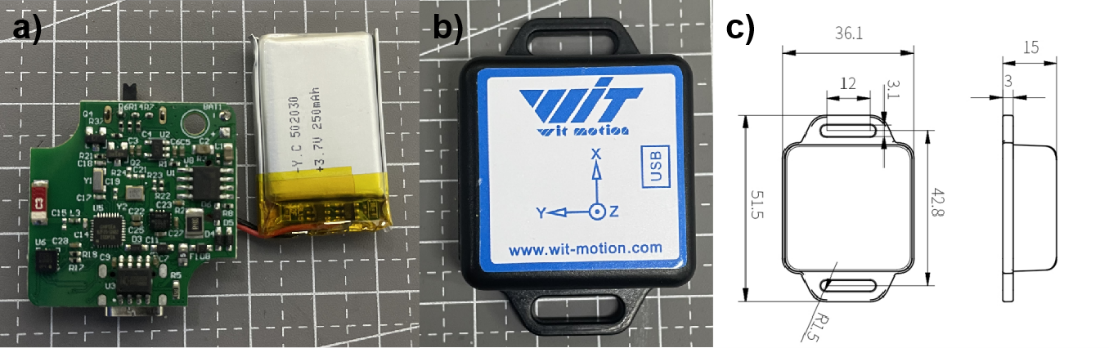


**Figure S10.** a) IMU circuit board photo. b) IMU physical photo. c) IMU housing dimensions.


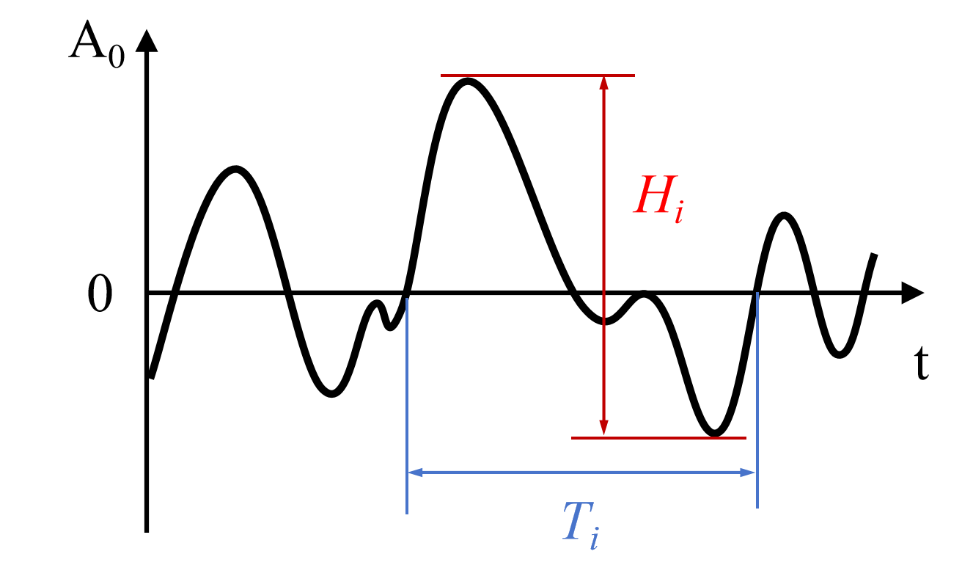


**Figure S11.** Schematic diagram of the zero-up crossing analysis method.


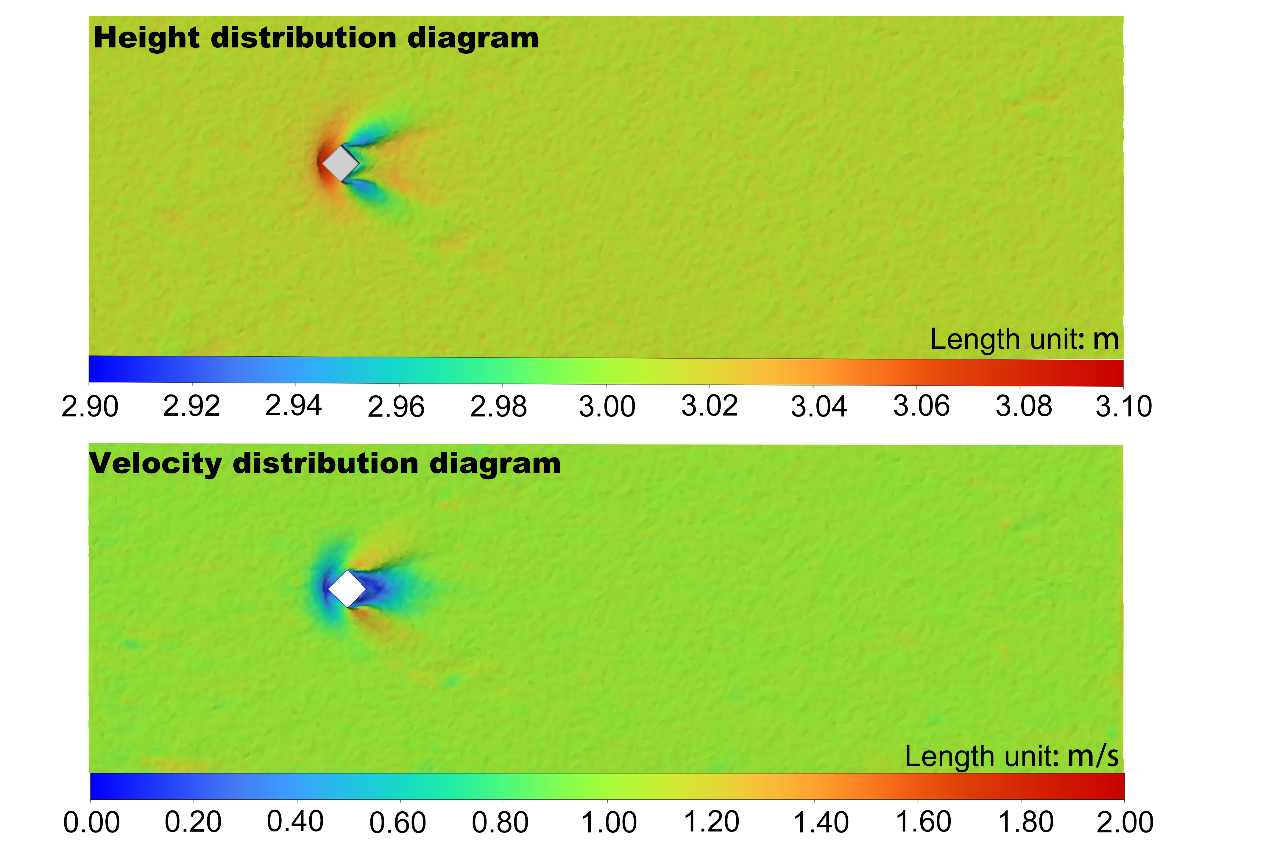


**Figure S12.** Height and velocity distribution daps derived from computational fluid dynamics (CFD) simulations of a cube shape.


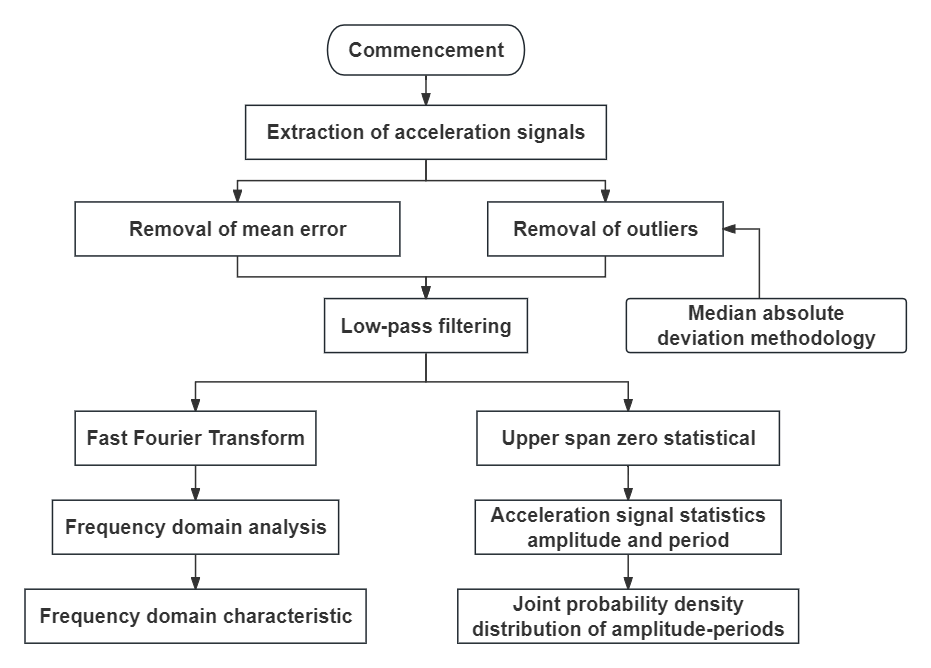


**Figure S13.** Flowchart of data processing.


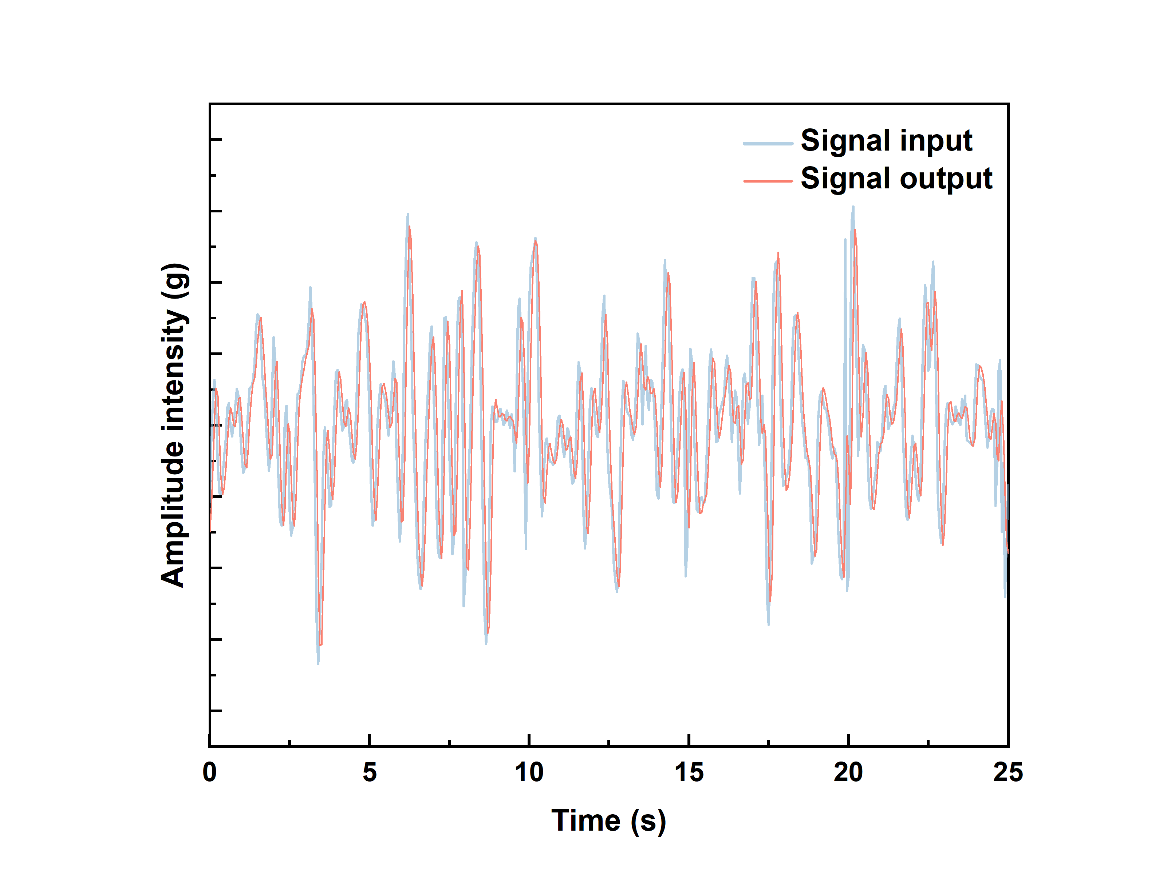


**Figure S14.** Comparison of filter input signal and output signal.

**
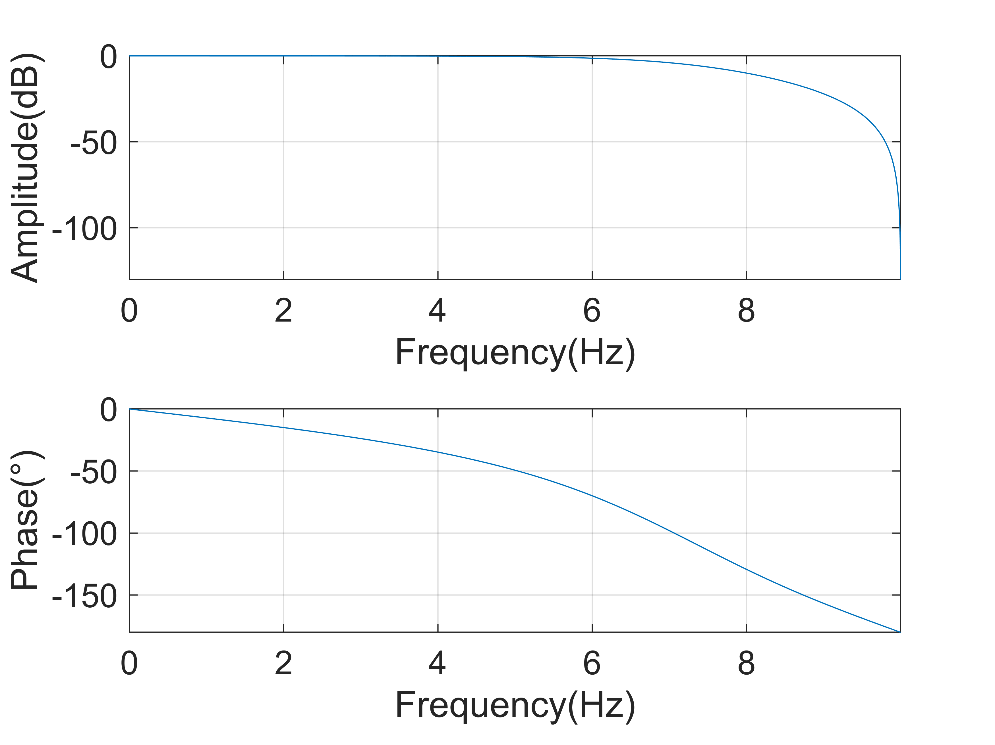
**

**Figure S15.** Butterworth low pass filter characteristic curve.


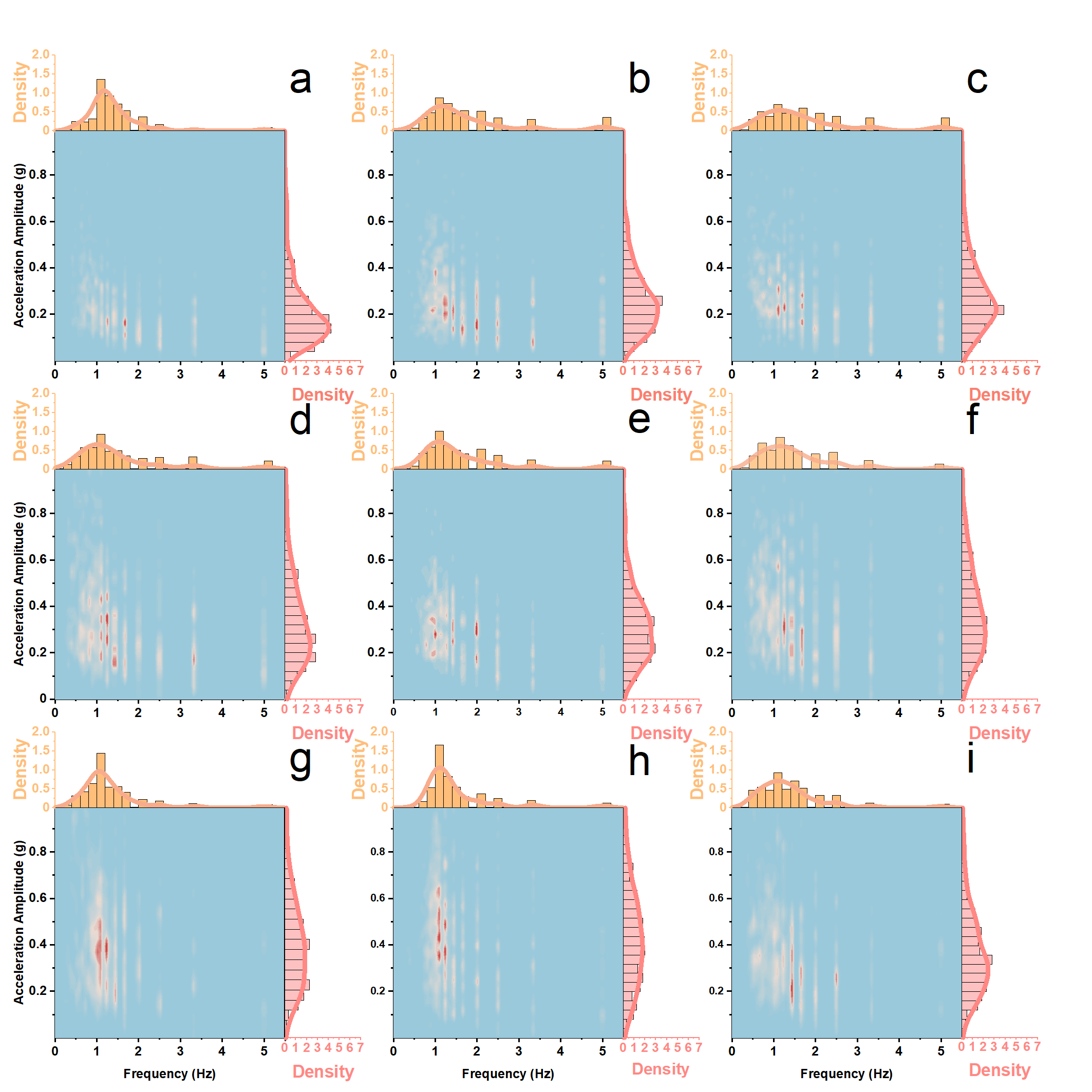


**Figure S16.** The Amplitude-Frequency joint distribution of different shaped shells.


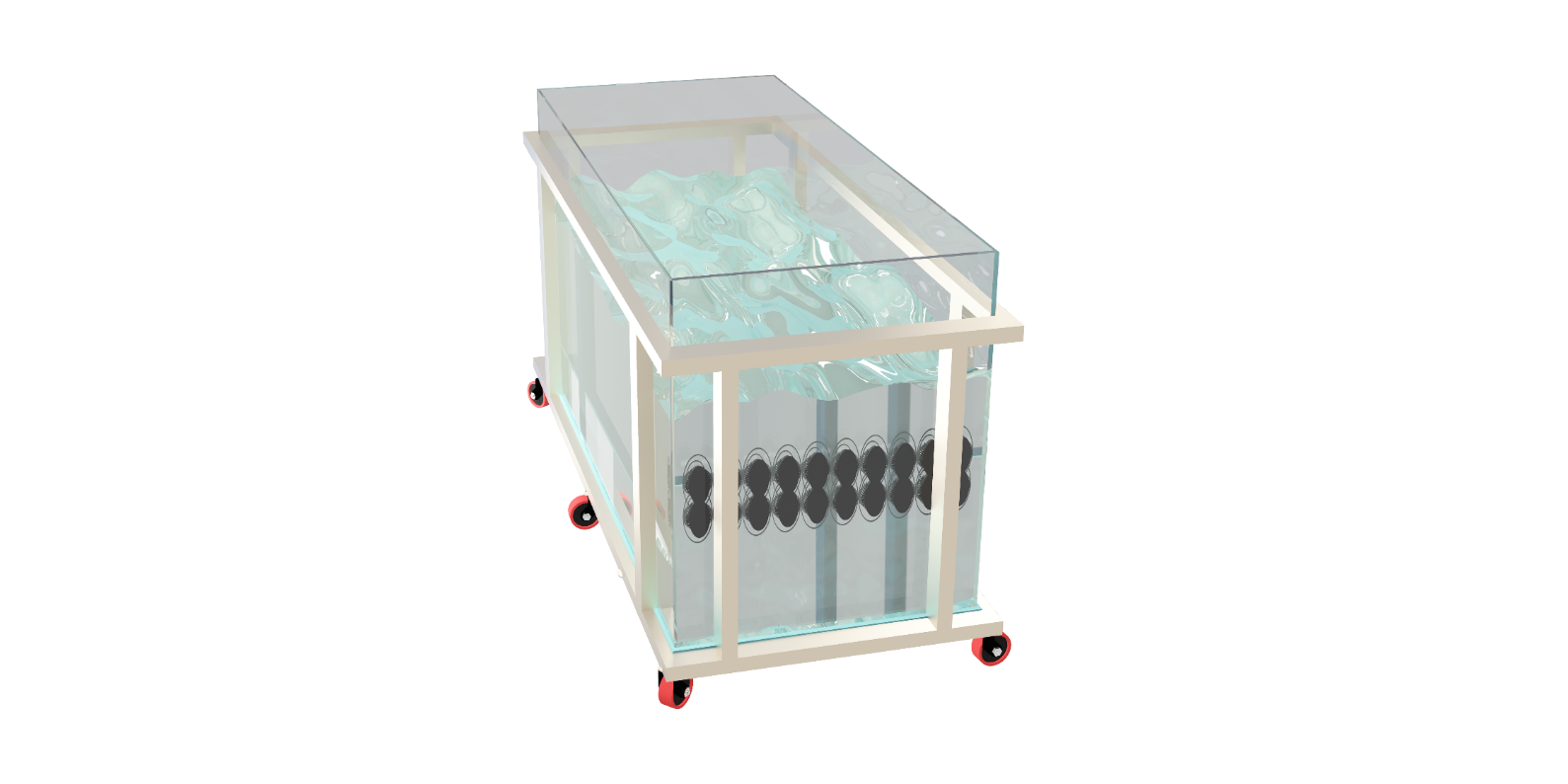


**Figure S17.** Laboratory wave pool configuration.

**Note S1. Interpretation of wave characterization parameters.**

In wave engineering, when counting wave spectral information, the height, period, frequency, and direction angle information of all experienced waves are often recorded in the statistical period. The waves are sorted in descending order from high to low according to the size of the wave height, and the characteristic parameters of the waves are defined as follows:

(1) Maximum wave height H_max_(m): It is to sort all the wave heights measured in the statistical period from the largest to the smallest and obtain the maximum value of the wave height, which is recorded as the maximum wave height; the corresponding period is recorded as the maximum wave period T_max_, and the corresponding frequency is recorded as the maximum wave frequency F_max_.

(2) Mean wave height H_mean_ (m): It is the average value of all wave heights measured in the statistical period from the largest to the smallest and is recorded as the mean wave height; its corresponding period is recorded as the mean wave period T_mean_, and its corresponding frequency is recorded as the mean wave frequency F_mean_.

(3) Significant wave height H_1/3_(m): The wave height obtained by sorting all the wave heights measured in the statistical period from the largest to the smallest and averaging the most significant wave heights in the first 1/3 is recognized as the significant wave height; its corresponding period is recognized as the significant period T_1/3_, and its corresponding frequency is recognized as the significant frequency F_1/3_.

(4) One-tenth wave height H_1/10_ (m): All wave heights measured during the statistical period are sorted from largest to smallest, and the average of the most significant wave heights in the first 1/10 is recorded as the giant wave height; its corresponding period is recorded as the one-tenth wave period T_1/10_, and its corresponding frequency is recorded as the one-tenth wave frequency F_1/10_.

Notably, most waves that can be observed with the naked eye have wave heights corresponding to the height of H_1/3_ in the characteristic parameter. This universal parameter can be regarded as the most probable wave height of the sea area, and its corresponding period T_1/3_ and frequency F_1/3_ can be considered as the most probable period and frequency of the sea area.

**Note S2. Wave-Body Interactions (WBI) principle.**

To describe the motion of the floating body and the effect of waves on it, we establish a coordinate system by choosing the central of gravity as the origin according to the characteristic of the Z-axis of the device under test passing through the central, as shown in Supplementary Figure S1 and Supplementary Figure S2. The plane z = 0 defines the mean free surface.

This object has a wet surface S that separates its interior from the water. Choose a surface element ***dS*** on the wet surface ***S*** as a reference point and construct a vector with the origin pointing to the reference point. Let ***U*** be the velocity vector at the reference point, then the velocity ***u*** of the surface element ***dS*** can be expressed as:

Where ***ω*** is the angular velocity vector corresponding to the rotation about the origin.

According to the potential-flow theory and fluid properties, the velocity potential ***Φ*** at any point on ***S*** should satisfy the following boundary conditions:

Where ***n*** is the normal flow rate of the fluid in contact with the face ***S***

Substituting Equation 3 gives the boundary conditions:

By further derivation, the forces applied under the six motion modes (j=1, 2, ...,6), as shown in Supplementary Figure S2, can be expressed as:

Here, ***p*** is the dynamic pressure of the fluid, ***ρ*** is the fluid density, and n_j_ are the individual components of the generalized normal vector ***n*** of the six modes of motion, as shown in Supplementary Figure S2, $\vec{n}$ expressed as follows:

From the many formulas above, we can see that the normal direction of the submerged surface of the float is the same as the fluid velocity near the interface, and the forces received in each axial direction of the float are strongly correlated with the normal direction of the submerged surface. It is further shown that the force received by the floating body is significantly axial.

**Note S3. The derivation process of Q_unit_ under ideal conditions.**

In order to better illustrate the problem of $Q_{unit}$ in the ideal state, we use a cylindrical floating body as shown in Figure S3 as the object of study to illustrate the specific form of its $Q_{unit}$. Since the dimensions of common Flo-TENG devices are much smaller than the wavelengths of actual sea waves, in practical applications, the wave forces acting on Flo-TENG devices are mainly reflected as wave drag force and inertial force, with the effect of the diffraction force being relatively small. In the Z-axis direction, the forces acting on the floating body primarily arise from the lifting effect of the waves, the body's own gravity, and buoyancy from the fluid. Hence, we can calculate the wave forces on the floating body model using the Morison equation, which is derived from potential flow theory. Taking the forces in the XOY plane as an instance, let's analyze the specific form of $\left| T_{r}\left( A_{m},\omega\right) \right|$ under ideal conditions.

The Morison equation is employed to account for both inertia and drag components that affect a structure exposed to wave action. Inertia forces are related to the acceleration of the water particles caused by the wave, while drag forces are related to the velocity of the water particles relative to the structure. In the context of Flo-TENG devices, which are typically much smaller than the wave lengths encountered, the Morison equation simplifies the analysis of forces exerted by relatively small-scale wave motions.

To determine $\left| T_{r}\left( A_{m},\omega\right) \right|$, we need to consider both the drag and inertia effects induced by the wave motion in relation to the device’s characteristics such as shape, size, orientation, and material properties. Thus, $\left| T_{r}\left( A_{m},\omega\right) \right|$ becomes a function encapsulating how efficiently the engaged Flo-TENG device can convert the wave's mechanical energy (accounting for aforementioned forces) into useful energy that can be transformed, reflective of the device's operational efficiency under specified wave conditions within the XOY plane. For the cylindrical model shown in Figure S3 the horizontal wave force on the unit column surface at any height z can be expressed as:

$f(z)=\frac{1}{2}C_{D}\rho Du(t)|u(t)|+C_{M}\rho\frac{\pi D^{2}}{4}a(t)$ Equation 8

In Equation 8, 𝑢(𝑡) and 𝑎(𝑡) represent the velocity and acceleration, respectively, of the water particles at height 𝑧, reflecting the action of the waves. 𝐶_𝐷_is the drag coefficient, and 𝐶_𝑀_ is the inertia coefficient. Both coefficients can be experimentally determined and are strongly related to the submerged shape of the floating body. 𝐷 represents the diameter of the cylinder, and 𝜌 is the density of the fluid. For simplicity, we will discuss the expression of the transfer function of this floating body using the simplest linear wave form. According to the linear wave theory, the horizontal velocity and acceleration of a monochromatic wave are given respectively by:

$u(t)=\frac{\pi H}{T}\frac{\cosh kz}{\sinh kh}\cos(kx-\omega t)$ Equation 9

$a(t)=\frac{2\pi^{2}H}{T^{2}}\frac{\cosh kz}{\sinh kh}\cos(kx-\omega t)$ Equation 10

Where, H represents the wave height, T is the wave period, k is the wavenumber, h is the water depth, ω is the angular frequency of the wave particles, and t is the propagation time. Consider the wavefront equation for a monochromatic wave as:

$H(t)=\frac{H}{2}\cos(kx-\omega t)$ Equation 11

Then Equation 9 and Equation 10 can be rewritten as:

$u(t)=\omega\frac{\cosh kz}{\sinh kh}H(t)$ Equation 12

$a(t)=\omega^{2}\frac{\cosh kz}{\sinh kh}H(t)$ Equation 13

The velocity spectral $S_{u}\left( \omega\right)$ and the acceleration spectral $S_{a}\left( \omega\right)$ can be obtained by comparison:

$S_{u}(\omega)=|Tr_{u}(i\omega)|^{2}S_{H}(\omega)=\omega\frac{\cosh kz}{\sinh kh}S_{H}(\omega)$ Equation 14

$S_{a}(\omega)=|Tr_{a}(i\omega)|^{2}S_{H}(\omega)=\omega^{2}\frac{\cosh kz}{\sinh kh}S_{H}(\omega)$ Equation 15

By comparison, the velocity spectral transfer function and the acceleration spectral transfer function of the water quality point are obtained:

$Tr_{u}(i\omega)=\omega\frac{\cosh kz}{\sinh kh}$ Equation 16

$Tr_{a}(i\omega)=\omega^{2}\frac{\cosh kz}{\sinh kh}$ Equation 17

According to the time-domain relation of Morrison's formula, the total horizontal force spectrum of a classical cylindrical floating body at height z and its transfer function can be derived through the above process after introducing the linearisation method of Borgman's drag force term as:

$\begin{matrix} S_{f(z)}(\omega)=\left(\frac{1}{2}C_{D}\rho D\sqrt{\frac{8}{\pi}}\sigma_{u}\omega\frac{\cosh kz}{\sinh kh}\right)^{2}S_{H}(\omega) \\ +\left(C_{M}\rho\frac{\pi D^{2}}{4}\omega^{2}\frac{\cosh kz}{\sinh kh}\right)^{2}S_{H}(\omega) \end{matrix}$ Equation 18 $Tr_{f(z)}(i\omega)=\sqrt{\left(\frac{1}{2}C_{D}\rho D\sqrt{\frac{8}{\pi}}\sigma_{u}\omega\frac{\cosh kz}{\sinh kh}\right)^{2}+\left(C_{M}\rho\frac{\pi D^{2}}{4}\omega^{2}\frac{\cosh kz}{\sinh kh}\right)^{2}}$Equation 19

In this context, $\boldsymbol{\sigma}_{\boldsymbol{u}}$ is the term introduced by Borgman's linearization method, representing the variance of the horizontal velocity of water particles. By integrating Equation 18 over the height range from $z_{1}$ to $z_{2}$, the total horizontal force spectrum of the submerged body within the horizontal plane and its transfer function for the depth range $z_{1}$ to $z_{2}$ can be obtained as follows:

$\begin{matrix} S_{F}(\omega)=\left(\frac{1}{2}C_{D}\rho D\sqrt{\frac{8}{\pi}}\frac{\omega}{\sinh kh}\int_{z1}^{z2} \sigma_{u}(z)\cosh kzdz\right)^{2}S_{H}(\omega) \\ +\left(C_{M}\rho\frac{\pi D^{2}}{4}\frac{\omega^{2}}{k}\frac{\sinh kz_{2}-\sinh kz_{1}}{\sinh kh}\right)^{2}S_{H}(\omega) \end{matrix}$Equation 20

$\begin{matrix} Tr_{f}(\omega)=\left|\left(\frac{1}{2}C_{D}\rho D\sqrt{\frac{8}{\pi}}\frac{\omega}{\sinh kh}\int_{z1}^{z2} \sigma_{u}(z)\cosh kzdz\right)^{2}\right. \\ \left.+\left(C_{M}\rho\frac{\pi D^{2}}{4}\frac{\omega^{2}}{k}\frac{\sinh kz_{2}-\sinh kz_{1}}{\sinh kh}\right)^{2}\right|^{\frac{1}{2}} \end{matrix}$ Equation 21

Considering that the effect of force on the device is an average effect, the magnitude and direction of the force at a given moment are determined. According to Newton's second law, the total horizontal force spectrum derived from the above equation differs from the actual acceleration spectrum of the device by a factor of the device's mass (m). From Equation 20 and Equation 21, we can observe that the transfer function is directly related to various physical quantities, including the shape of the device, fluid density, and immersion depth. Thus, the $Q_{unit}$ evaluation method proposed in this paper and the impact of different experimental parameters on $Q_{unit}$ demonstrate scientific validity and reasonableness in assessing the energy absorption and swaying behavior of the Flo-TENG casing.

**Note S4.** **Calculation method of the center of gravity.**

The object under test used in the experiment consisted of an IMU unit, a shell structure printed in R4600 resin material, a number of counterweight blocks with threads (***w_1_***,***w_2_***,***w_3_,***...,***w_n_***), and waterproof adhesive, as shown in Supplementary Figure S9.

We record the mass of the overall device under test as *M_c_*, the center of gravity as oc, the mass of the shell as *M_b_*, the center of gravity as *O_b_* (the center of gravity of the shells with different shapes is given by Supplementary Table S2), the mass of the IMU unit as M_IMU_, the center of gravity as *O_IMU_*, and the masses and center of gravity of several counterweights are noted as *M_w_* = [*M_w1_*,*M_w2_*,*M_w3_*,...,*M_wn_*] and *O_w_* = [*O_w1_*,*O_w2_*,*O_w3_*,...,*O_wn_*], in turn.

Here, the center of gravity is calculated using the horizontal plane on which the device is placed as the reference plane and the mass is weighed by an electronic scale.

The formula for calculating the center of gravity of the overall device can be expressed as:

Using Equation 8, we can calculate the position of the center of gravity and the position of the counterweight block for different test objects, and screw the threaded counterweight block to the specified height of the support bar in accordance with the required center of gravity position for the experiment.

**Note S5. Test principle of inertial measurement unit (IMU).**

An inertial measurement unit (IMU) is a device that measures an object's three-axis attitude angle (or angular rate) as well as acceleration. Typically, an IMU contains three single-axis accelerometers and three single-axis gyroscopes and magnetometers and other sensors, as shown in Supplementary Figure S10a. Based on these sensors, the IMU can convert the motion signals of the measured object into electrical signals and, further, into digital signal outputs. The operation of the accelerometer inside the IMU depends mainly on the effect of the motion of the object to be measured on its internal variable capacitor or variable resistor. These changes will be converted into electrical signals by a special processing chip and further processed for output as digital signals. In use, we need to calibrate the IMU by placing it flat on a horizontal surface. The calibration is successful when the output of the sensor signal is 0 g, 0 g and 1 g (g = 9.8 m/s^2^) in x, y and z axes respectively. The BWT901BLECL5.0 from Wit Motion features small dimensions (51.5 mm $\times$ 36.1 mm $\times$ 15 mm, as shown in Supplementary Figure S10b and Supplementary Figure S10c), a large sampling frequency (up to 200Hz), low power consumption (3.7, DC, 15 mA), and the ability to use Bluetooth 5.0 transmission protocol. The main operating parameters of this IMU, as shown in Supplementary Table.S1. The accelerometer range is ±16 g with a resolution of 0.005 (g/LSB). According to the quist sampling theorem, the sensor can be used to acquire acceleration signals from floating objects with a response frequency of less than 10 Hz.

**Note S6. Calculation of frequency and amplitude of shell motion.**

Since real ocean waves are formed by the superposition of multiple waves of different frequencies and have randomness, in a complete wave process, the wave height will produce multiple high and low states as the wave travels. Based on such a characteristic of the waves, we regard the wave height rise and fall process of two times across the zero point as a complete wave process, i.e., the zero up-crossing analysis method.

On the basis that the action of waves on the Flo-TENG shell has synchronization with the travel and rise and fall of waves, in this paper, the statistics are carried out using the zero up-crossing analysis method for the wave heights and frequencies as well as for the amplitude and frequency of the motion of the Flo-TENG, as shown in Supplementary Figure S11.

Consider H_i_ as the amplitude of the signal under study, *T_i_* as the period, and take the reciprocal of *T_i_* to get the frequency *F_i_*. then the amplitude, period and frequency information are intervalized to get their probability density histograms. The maximum entropy distribution is fitted to the amplitude and the Gaussian kernel fitting method is fitted to the frequency to obtain the probability density histograms and probability density distribution curves for each axis shown in this paper.

**Note S7. the maximum-entropy fitting method.**

In accordance with the principle of maximum entropy, as long as the objective constraints can be determined, the distribution function solved should be able to describe the data of the acceleration samples well. Through the analysis of the relationship between waves and acceleration, both should follow the same probability distribution. These distributions can be derived from Rayleigh or Weibull distributions under different wave environments with the same constraints based on long time observations.

Real ocean waves can be regarded as a combination of two distributions. To characterize the distribution of waves and acceleration better and avoid the influence of distribution model selection, this paper chooses the maximum-entropy distribution function that can effectively describe the characteristics and guide the selection of distribution type through parameter calculation.

For accelerated samples denoted as A=[a_1_,a_2_,a_3_,...,a_n_], the distribution function and probability density function are given by the following equation:

where $\left( \alpha,\beta,\gamma,A_{0} \right)$ are the four fitting parameters, where $A_{min}\geq A_{0}>0$, $A_{max}$ is the maximum value of the acceleration sample, $A_{min}$ is the minimum value of the acceleration sample, $A_{0}$ is the fitted location parameter of the distribution function, and $\Gamma\left( x \right)$ is the gamma function.

Clearly, this maximum entropy distribution contains four fitting parameters $\left( \alpha,\beta,\gamma,A_{0} \right)$ that need to be estimated based on the sample. Here we use the three-parameter method to estimate the sample parameters, and this parameter estimation method involves the following sample eigenvalues: mean ($\bar{A}$), standard deviation (σ), coefficient of variation (Cv), coefficient of skewness (Cs), and coefficient of kurtosis (Ce). The formulas for calculating these eigenvalues are as follows:

The mean, standard deviation, and coefficient of variation indicate the concentration trend of the distribution; the skewness coefficient indicates the degree of asymmetry of the distribution; and the kurtosis coefficient indicates the relative number of variables distributed near the mean or the plurality.

The basic steps of the three-parameter method of moments used in this paper are as follows：

1. Determine the value $A_{0}$ in the maximum entropy distribution.

2. The nonlinear relationship between the sample eigenvalues ($\bar{A}$, C_v_, C_s_) and the fitting parameters (α, β, γ) is obtained by solving for the 0th, 1st, 2nd, and 3rd order moments of origin of the distribution function *P* and the sample moments of origin (shown Equation 16, Equation 17 and Equation 18).

3. Finally, iterate through all the conforming $A_{0}$ values, and determine the best-fitting parameters by testing the degree of fit through the K-S method.

**Note S8. Computational Fluid Dynamics (CFD) Simulation**

This study conducts three-dimensional numerical simulations of the motion of various floating objects used in experiments using Computational Fluid Dynamics (CFD). We carried out transient simulations of the fluid-structure interaction between the floating objects and water waves at the same flow velocity (V=1 m/s), actual dimensions, material density, and rotational inertias along each axis.

The simulation results vividly demonstrate that changes in flow direction and velocity after interaction with the floating objects are closely related to the shape of the floating object's hull. The horizontal force on the cube is greater than that on the sphere, yet the wave accumulation in front of the wavefront forms a layer of fluid deceleration zone, making it more difficult for the cube to oscillate.

**Note S9. Limitations and significance of comparisons between laboratory waves and natural waves.**

At present, researchers frequently employ laboratory-generated waves with a frequency of 0.5 Hz to assess the performance of the Flo-TENG device during the design phase. The principal objective of the comparative analysis experiment on laboratory waves and actual waves in this article is to identify the similarities and differences in the effects of artificially generated waves in the laboratory and real ocean waves on the same device. By conducting a comparative analysis of the two types of waves, the differences between laboratory-generated waves and real ocean waves can be elucidated, thereby providing guidance for simulating waves in the limited laboratory environment based on wave spectrum parameters to more accurately reflect the characteristics of ocean waves. Although this paper attempts to maintain the similarity of the two wave environments according to the froude criterion in the comparative analysis experiments between laboratory water waves and natural ocean waves, the data are still limited due to the experimental conditions such as the size of the laboratory wave pool.

Undoubtedly, the development of Flo-TENG at its current stage faces the challenge of significant differences between the outputs obtained in an ideal laboratory wave environment and those in a real ocean wave environment. The laboratory-generated waves in the current stage are mainly basic and allow for accurate quantification and reproducibility. They are essential for driving theoretical output simulations of Flo-TENG and measuring the device's inherent frequency. The test results under laboratory-generated waves provide cross-comparability for Flo-TENG and play an irreplaceable role in measuring the parameters of high-performance Flo-TENG. On the other hand, ocean waves are random, constantly changing, and unable to be completely replicated, representing the environmental excitation that the device will actually encounter during operation. In practical applications, the output of Flo-TENG under ocean wave excitation represents the electric power that is effectively converted during actual use. This is also the primary indicator that needs to be optimized and improved when designing high-performance Flo-TENG systems. Therefore, analyzing the similarities and differences between real ocean waves and laboratory-generated waves, identifying the significant wave frequencies and the distribution range of energy with respect to frequency in real ocean waves, and replicating waves in the laboratory that possess these characteristics hold great importance in narrowing the gap between theoretical and actual outputs. While the measured motion data of Flo-TENG in the two environments may differ significantly, both are indispensable. Many research efforts are also attempting to bridge the gap between the two. Just like the differences between first-principles calculations in the field of materials and the actual properties of materials, The results of first-principles calculations are obtained under ideal conditions at 0 K. However, the measurement of material properties in reality is limited by physical constraints, making it impossible to reach a testing environment at absolute zero. This limitation leads to significant differences between the theoretical calculations and the actual properties of materials. Indeed, we cannot deny the significance and contributions of both theoretical calculations and experimental measurements in researching and understanding material properties. In the writing and revision of this manuscript, we are also aware of the similarities and differences between the measured data of laboratory-generated waves and real ocean waves. Just like in other fields, we are constrained by the limitations of the experimental environment and the scarcity of data, and therefore unable to bridge the gap between the two in a short period of time.

Through frequency domain analysis of real waves and lab-generated wave signals, our work has revealed the similarities, differences, and numerous patterns between the two. The discoveries made in our manuscript hold the potential to provide guidance and reference for the simulation of random wave in laboratory settings. Although it is not currently feasible to completely replicate waves with oceanic characteristics in a lab within a short period of time, these discussions in the paper also offer some insights into bridging the gap between the theoretical design output and the actual output of Flo-TENG.

**Note S10. Bias Removal.**

After the manual installation and fixation of the IMU and each counterweight block, there may be slight differences or positional offsets among different devices. If there is a subtle deviation from the center of gravity, this will cause the acceleration component on the Z-axis to be distributed to the X-axis or Y-axis, introducing unnecessary errors to the acceleration measurement. The added or subtracted value is due to the original positional offset, which is a fixed constant known as bias. Based on this characteristic, the changes in acceleration should fluctuate around this constant. In order to better compare the motion characteristics of each axis of the devices and mitigate the inconvenience caused by bias, we subtract the mean value from the acceleration data of each axis, i.e., remove bias, so that the final acceleration curve fluctuates around the zero mark, facilitating our subsequent data processing and comparison.

**Note S11. Outlier Data Processing Method.**

During actual sea wave testing, in order to prevent the devices from being lost, a fishing line is used to secure the devices within a certain range. Occasionally, when the device experiences significant wave impacts and is about to leave the designated range, there will be an instant traction from the fishing line. This force can cause a sudden change in the device's acceleration signal, resulting in multiple outliers in the measured signal that deviate from the normal amplitude range. To mitigate this error introduced by the real-world scenario, we employ the median absolute deviation (MAD) method to replace such values with the median of the adjacent forty points, ensuring the reliability of the signal.

**Note S12. Filtering Method.**

Although the IMU output data has been filtered using a Kalman filter, there are still defects and high-frequency noise in the waveform components. In order to better reflect the interaction between the Flo-TENG and the random water waves in real sea conditions, it is necessary to filter out noise that is higher than the wave excitation frequency and hull oscillation frequency. Here, we employ Butterworth low-pass filters and moving average filters to reduce these defects, as shown in Supplementary Figure S14. The characteristics of the Butterworth low-pass filter used are demonstrated in Supplementary Figure S15.

**Note S13. Kolmogorov-Smirnov Test (K-S Test).**

To better assess the adequacy of a maximum entropy distribution in representing a sample, this study employs the Kolmogorov-Smirnov (K-S) test method. Let the population distribution function be denoted as P(a) and the theoretical distribution function be P_0_(a). The K-S hypothesis test question is as follows:

The physical interpretation is that when P(a) follows the theoretical distribution function P_0_(a), the hypothesis is accepted and denoted as 0; otherwise, the hypothesis is rejected and denoted as 1. For a dataset, A=[a_1_,a_2_,a_3_,…,a_n_], iterate through all sample points and calculate their cumulative frequency function P_n_(a). Furthermore, under the assumption of A_0_, compute the observed value P_0_(a) for each observed sample and calculate the K-S statistic D_n_ using the following formula:

Where i = 1, 2, …, n. Set the significance level = 0.05 and calculate the critical value D_n_(α). If D_n_ > D_n_(α), reject the hypothesis; otherwise, accept the hypothesis. In this case, it indicates that the sample distribution function has a 95% chance of following the theoretical distribution function, demonstrating a good fit for the function.

**Table S1. Main operating parameters of the IMU**

| Sensor | Parameters | Value |
| --- | --- | --- |
| Accelerometer | Range | ±16 g |
|  | Resolution | 0.005 (g/LSB) |
|  | RMS noise | 0.75~1 mg~rms |
|  | Static zero floating | ±20~40 mg |
|  | [Temperature drift](https://context.reverso.net/%E7%BF%BB%E8%AF%91/%E8%8B%B1%E8%AF%AD-%E4%B8%AD%E6%96%87/temperature+drift) | ±0.15 mg/℃ |
|  | Bandwidth | 256 Hz |
| Gyroscopes | Range | ±2000 °/s |
|  | Resolution | 0.061 (°/s)~rms |
|  | RMS noise | 0.028~0.07 (°/s)~rms |
|  | Static zero floating | ±0.5~1 °/s |
|  | [Temperature drift](https://context.reverso.net/%E7%BF%BB%E8%AF%91/%E8%8B%B1%E8%AF%AD-%E4%B8%AD%E6%96%87/temperature+drift) | ±0.005~0.015 (°/s)/℃ |
|  | Bandwidth | 256 Hz |

**Table S2. Centroid position of different models**

| Shape | X(mm) | Y(mm) | Z(mm) |
| --- | --- | --- | --- |
| Shaped prism | 0.0000 | 6.1026 | 24.5727 |
| Quadrangular column | 0.0000 | 0.0000 | 18.2126 |
| Four-pronged table | 0.0000 | 0.0000 | 25.2646 |
| Round table | 0.0000 | 0.0000 | 26.1328 |
| Hemispheres | 0.0000 | 0.0000 | 28.8480 |
